# Unveiling the Dynamic Self-Assembly of a Recombinant Dragline-Silk-Mimicking Protein

**DOI:** 10.1021/acs.biomac.3c01239

**Published:** 2024-02-12

**Authors:** Dongqing Wu, Anamaria Koscic, Sonja Schneider, Romeo C. A. Dubini, Diana C. Rodriguez Camargo, Sabine Schneider, Petra Rovó

**Affiliations:** † Department of Chemistry, Faculty of Chemistry and Pharmacy, Ludwig-Maximilians-Universität München, 81377 Munich, Germany; ‡ Center for Nanoscience (CeNS), Faculty of Physics, Ludwig-Maximilians-Universität München, 80799 Munich, Germany; § Institute of Science and Technology Austria, 3400 Klosterneuburg, Austria

## Abstract

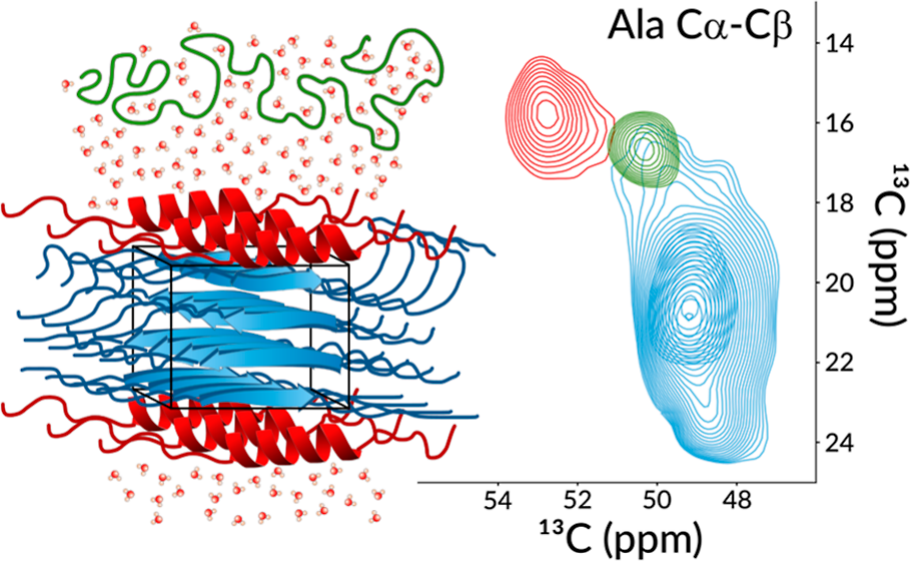

Despite the considerable interest in the recombinant production of synthetic spider silk fibers that possess mechanical properties similar to those of native spider silks, such as the cost-effectiveness, tunability, and scalability realization, is still lacking. To address this long-standing challenge, we have constructed an artificial spider silk gene using Golden Gate assembly for the recombinant bacterial production of dragline-mimicking silk, incorporating all the essential components: the N-terminal domain, a 33-residue-long major-ampullate-spidroin-inspired segment repeated 16 times, and the C-terminal domain (N16C). This designed silk-like protein was successfully expressed in *Escherichia coli*, purified, and cast into films from formic acid. We produced uniformly ^13^C–^15^N-labeled N16C films and employed solid-state magic-angle spinning nuclear magnetic resonance (NMR) for characterization. Thus, we could demonstrate that our bioengineered silk-like protein self-assembles into a film where, when hydrated, the solvent-exposed layer of the rigid, β-nanocrystalline polyalanine core undergoes a transition to an α-helical structure, gaining mobility to the extent that it fully dissolves in water and transforms into a highly dynamic random coil. This hydration-induced behavior induces chain dynamics in the glycine-rich amorphous soft segments on the microsecond time scale, contributing to the elasticity of the solid material. Our findings not only reveal the presence of structurally and dynamically distinct segments within the film’s superstructure but also highlight the complexity of the self-organization responsible for the exceptional mechanical properties observed in proteins that mimic dragline silk.

## Introduction

Spider dragline silks, or spidroins, are natural fibers with exceptional properties including their unique lightweight, impressive extensibility, high tensile strength, durability, and biocompatibility.^1−7^ These unparalleled qualities make spidroins valuable materials for various industrial and biomedical applications. Throughout history, they have been utilized in diverse fields such as clothing, fishing, painting, medicine, and weaponry.^4,8^ Despite significant efforts in replicating the mechanical features of spidroins, artificial fibers often fall short in terms of versatility and overall performance compared to those of natural spider silks.^9−11^ However, recent advancements in producing spidroins with previously unattainable molecular weights through heterologous bacterial expression^12^ or utilizing transgenic silkworms^13^ are bringing us closer to the commercialization of tailor-made bioengineered silk fibers. Spider dragline silk has attracted considerable attention due to its exceptionally high tensile strength, exceeding that of steel, and toughness that is three times higher than that of Kevlar.^4,14^ Most dragline silks are made from two major ampullate spidroin proteins [major ampullate spidroin protein 1 (MaSp1) and major ampullate spidroin protein 2 (MaSp2)], which share a common structure, characterized by a low-complexity, highly repetitive core with up to a few hundred repeats of 20–200 amino acids. This core is flanked by highly conserved, nonrepetitive α-helical N- and C-terminal domains (NTD and CTD). The repeat regions have a block copolymer structure in which hydrophilic “soft” glycine (Gly)-rich and hydrophobic “hard” alanine (Ala)-rich segments alternate. These segments are composed of shorter consensus motifs, including poly(A), poly(GA), GGX, GSG, QQ, and GPGXX stretches, where X = Y (Tyr, tyrosine), L (Leu, leucine), or Q (Gln, glutamine).

Solid-state nuclear magnetic resonance (NMR) data suggested that the Gly-rich segments predominantly adopt semiextended 3_1_-helices, while the GPGGX and GPGQQ elements of MaSp2 form elastin-like type II β-turns.^15−24^ Alternatively, these segments can be incorporated into β-sheet structures, and thus, they are part of the hard segments.^19,20,24,25^ The low density of hydrogen bonds in the soft Gly-rich regions grants extensibility to the dragline fiber,^7,26^ while the successive β-turns are associated with the supercontractive property of the silk fiber.^21^ Upon sheer-induced stress, the majority of the alanine residues arrange into β-sheets and form β-nanocrystals, whereas the alanines in poly(GGA) adopt 3_1_-helix structures.^15,18,24,25^ In the β-nanocrystals, the poly(A) β-sheets tightly interlock, resembling the steric zippers of amyloid crystals, thereby impeding water penetration between the poly(A) sheets.^17^ The relatively small crystalline domains^27−31^ embedded within the semiamorphous matrix act as intermolecular cross-links, contributing to the high tensile strength of the spun fibers.^7,24,26^ Additionally, the large number of repeated core units enhances interchain interactions and reduces chain-end defects, further augmenting the unprecedented tensile strength of the silk fibers.^7,12,13,32^

The importance of the high repeat number in recombinant spider silk proteins was recognized early on,^33−35^ and attempts have been made to increase the repeat number all the way up to 192.^12,32^ Achieving such a feat required the implementation of extensive metabolic engineering and synthetic biology approaches, which have not yet been adapted for large-scale biotechnological production. In addition to the repeat number, the terminal domains play fundamental roles in determining the properties of silk proteins, especially with respect to protein gland solubility and initiation of fiber assembly through salt- and pH-dependent dimerization.^36−38^ Previous studies have established a correlation between recombinantly produced silk’s tensile properties, such as the Young’s modulus, strength, and toughness of the fibers, with the presence of the CTD and the NTD.^9,39^ For such reasons, the inclusion of the highly conserved globular terminal domains in the final spidroin product is necessary, especially when assembly and solubility properties are of interest.^36^

Repetitive DNA sequences provide challenges for standard cloning techniques, for example, due to their inherent ability to recombine and hence instability. Typically, recombinant tandem-repeat DNA sequences of fibrous proteins, including mimics of spider silks (reviewed in refs (5, 11, 40–42)), collagen,^43^ elastin,^44,45^ keratin,^46^ and resilin,^47^ are constructed via stepwise concatenation,^48,49^ recursive directional ligation,^12,44,45^ or step-by-step directional approaches,^50−52^ which involve several rounds of plasmid amplification, digestion, ligation, and possible sequencing between repeat extensions.^52−54^ In these stepwise techniques, at each oligomerization step, the cloning efficiency is greatly reduced by the possibility of obtaining empty vectors or self-ligated and circularized inserts. Aside from the time and material costs associated with these traditional approaches, a major drawback of a few of these methods is the scars that remain at the recombinant sites in the final constructs. These scars are then translated into extraneous amino acids in the primary protein sequence, compromising the accuracy and potentially the structural integrity of the expressed protein. Nevertheless, seamless stepwise cloning has been successfully applied in a few instances to produce recombinant fibrous proteins.^44,55−59^

**Figure 1:**
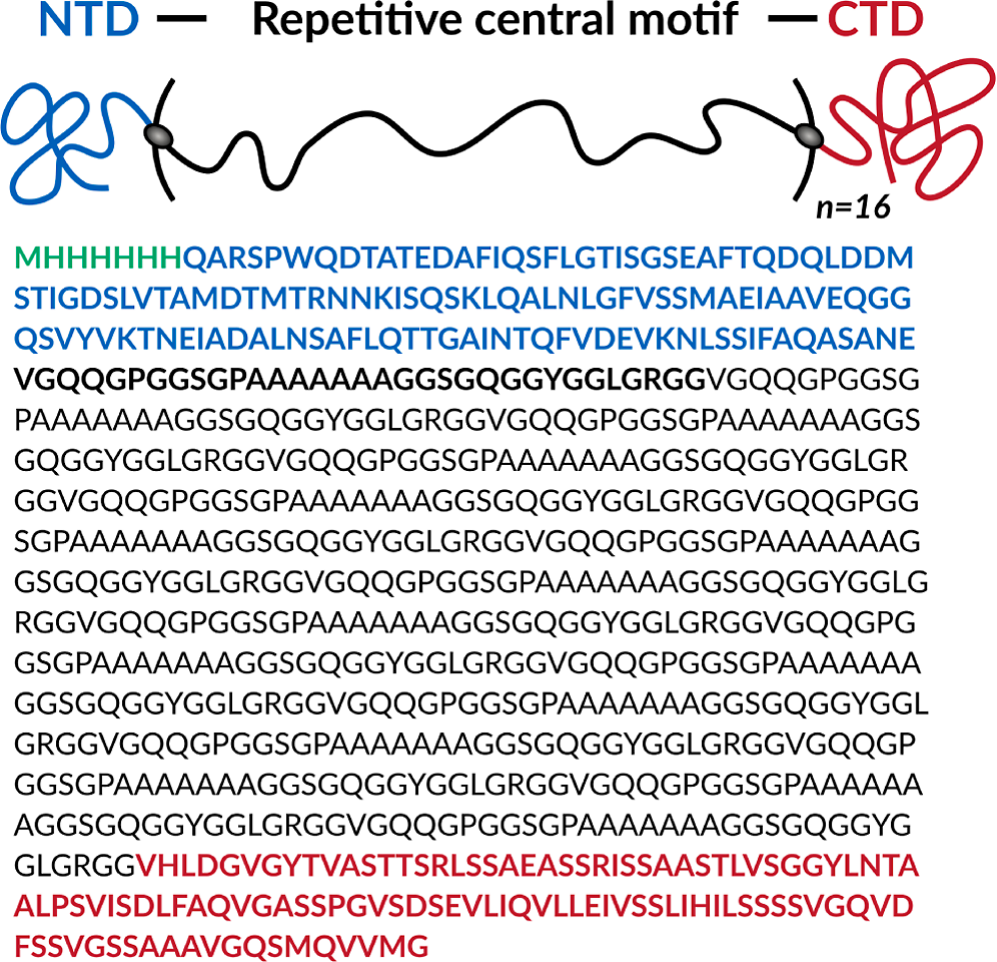
Schematic representation and amino acid sequence of the N16C protein construct: NTD (blue), derived from the *N. clavipes* MaSp2, the repetitive central domain (S16, black), based on *N. clavipes* MaSp1 and MaSp2, and CTD (maroon) from *N. clavipes* MiSp1. Amino acids labeled with green account for the His-tag used for purification purposes.

To overcome these shortcomings, in this study, we used Golden Gate assembly^60^ to generate an expression construct for a spidroin mimic, called N16C (Figure 1), which includes both the N- and C-terminal domains and 16 repeats of the repetitive core units. Golden Gate assembly relies on type II restriction endonucleases, which cut double-stranded DNA outside their recognition sequence and leave a short, single-stranded, user-defined overhang that guarantees ordered gene assembly of multiple constituents. The restriction sites are eliminated during subcloning, which allows for simultaneous digestion and ligation in a one-pot reaction and facilitates the seamless assembly of gene constituents. Previously, Golden Gate assembly has been successfully used to straightforwardly assemble repetitive DNA sequences encoding elastin-like proteins (ELPs).^59^

High repeat numbers and the presence of terminal domains are necessary but are not sufficient requirements for reproducing the properties of natural silk fibers. The failure in natural silk reproduction arises from the incomplete understanding of the driving forces behind the protein’s self-assembly across multiple spatial scales. Open questions include the exact structures of the involved molecules and sequence motifs, the types of interactions and transformations they undergo, the kinetics and thermodynamics of their interactions, and the dynamics that affect the local energetics and the macroscale mechanics of the silk fibers. Addressing these questions requires atomic-level insights into the protein assembly both experimentally and computationally. Solid-state magic-angle spinning (MAS) NMR spectroscopy is arguably the most suitable experimental technique to investigate complex heterogeneous systems as it has been demonstrated for natural^17−21,23−25,61−65^ and genetically engineered spider silks,^4,56,66−70^ or for selectively isotope-enriched silk-mimicking model peptides as reviewed in refs (71–74).

To fully exploit the advantages of recombinant spider silk production, we expressed and purified a uniformly ^13^C–^15^N-labeled version of our designed spidroin mimic N16C and measured its solid-state MAS NMR spectra to gain insights into the local structure, hydrogen-bonding, nanosecond and microsecond time-scale dynamics, and hydration-induced macroscopic organization of each amino acid types in the cast film. Fast MAS (55.55 kHz) and high magnetic fields (700 MHz) combined with proton detection significantly improved the sensitivity and the resolution of the acquired spectra, allowing for the almost complete amino-acid-specific assignment of the repetitive core, which then facilitated the analysis of site-specific dynamics across multiple time scales. The uncovered correlation between the atomic-level structure and hydration-induced local dynamics of the designed synthetic spidroins provides critical insight into the dynamic organization of natural silks and engineered silk-like proteins.

## Materials and Methods

### Materials

Chemicals and primers were purchased from Sigma-Aldrich. Restriction enzymes and DNA purification kits were purchased from New England Biolabs (Ipswitch, MA). Plasmids and vectors were purchased from Genscript (Piscataway, New Jersey). ^13^C-labeled glucose and ^15^N-labeled ammonium chloride used for the production of the isotope-enriched protein samples for NMR studies were purchased from Eurisotop (Germany). Double-distilled water was collected from a Millipore system.

### Cloning of Expression Constructs

**Figure 2:**
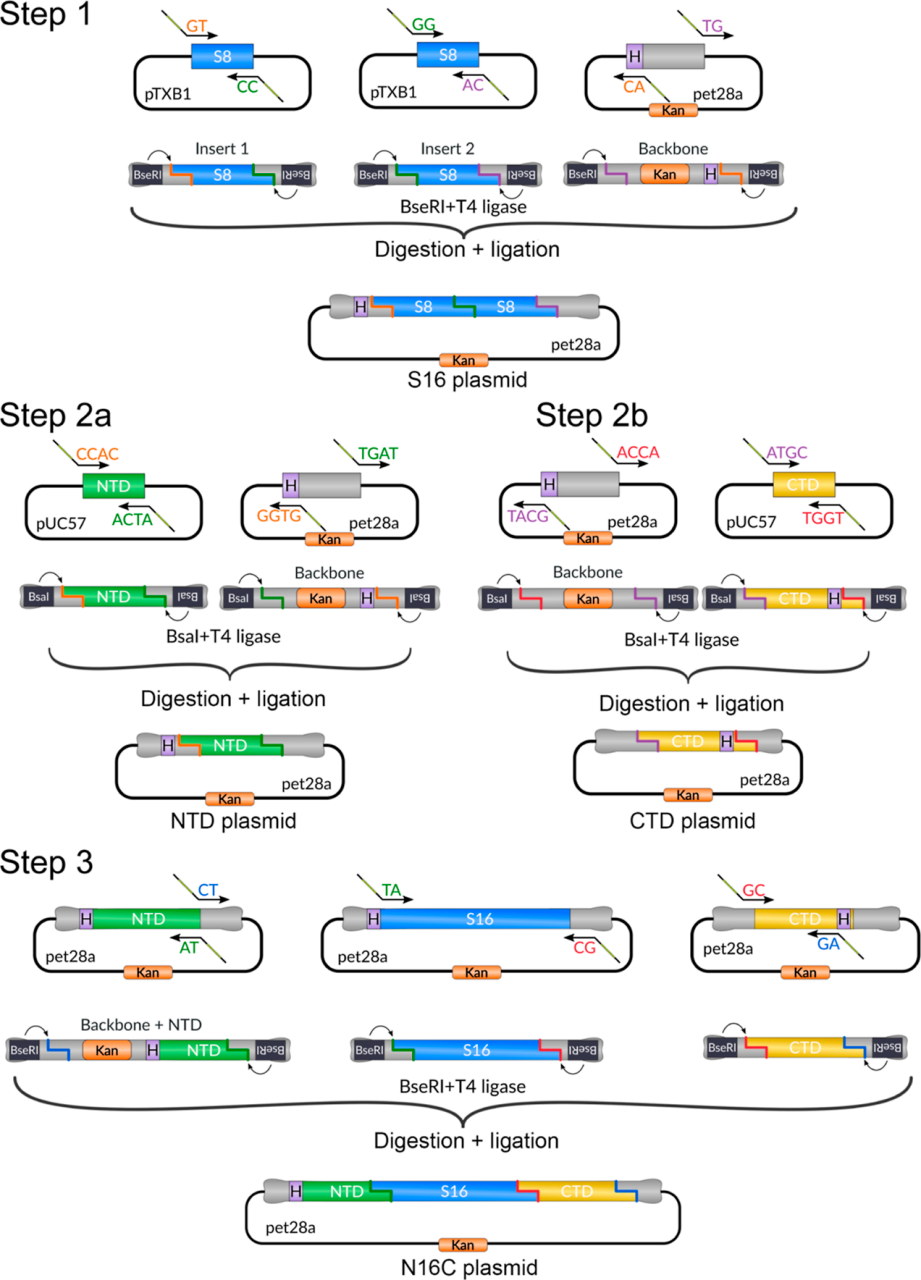
Golden Gate assembly of S16, NTD, CTD, and N16C. The construction of S16 and N16C used the type IIs restriction endonuclease *BseR*I and that of NTD and CTD *Bsa*I. The PCR-amplified linear products are digested and ligated in a single one-pot reaction leading to an ordered seamless assembly of modules without restriction sites. Steps 1, 2b, and 2b create the pET28a-based expression vectors for S16, NTD, and CTD, and in Step 3, these vectors are used to construct N16C. The backbone pET28a vector contains a kanamycin resistance gene (Kan), an N-terminal His-tag, and a T7-promoter region.

Overall four different expression constructs for the heterologous expression of *Nephilia clavipes* derived from the major and minor ampullate spidroin proteins, MaSp4 and MiSp2, in *Escherichia coli* were assembled with Golden Gate cloning: the NTD with an N-terminal hexahistidine-tag (His-tag), the CTD with a C-terminal His-tag, the repetitive core containing 16 repeats with an N-terminal His-tag (S16), and finally, the fully assembled construct consisting of the His-tagged NTD, S16 core, and the CTD (N16C). The whole Golden Gate assembly workflow is shown in Figure 2. For the assembly of the core domain, the synthetic gene purchased from GenScript encoding 8 repeat units and codon-optimized for *E. coli* expression was cloned into a pTXB1 vector. One oligonucleotide monomer building block was 99 nucleotides long and encoded 33 amino acids (VGQQGPGGSGP**AAAAAAA**GGSGQGGYGGLGRGG); the β-sheet-forming polyalanine domain is in bold. The codon-optimized versions of the genes encoding the NTD and CTD, which were derived from *N. clavipes* MaSp4 (GenBank: MF955711) and MiSp1 (GenBank: MF955722), respectively, were cloned into pUC57 vectors (GenScript).

For the construction of the linearized modules used in the Golden Gate cloning, primers were designed to contain either a *BseR*I or a *Bsa*I recognition site. All primers are listed in Table S1. The backbone for the Golden Gate assembly was polymerase chain reaction (PCR)-amplified from the pET28a plasmid. Here, primer annealing was achieved by decreasing the temperature in increments of 0.1 °C/s from 98 to 70 °C and incubation at 70 °C for 30 s. This was followed by an extension period at 72 °C for 30 s per kb with Taq DNA polymerase and strand separation at 98 °C for 10 s. This cycle was repeated 35 times. The final extension was performed at 72 °C for 10 min and then kept at 4 °C. To avoid mispriming during PCR, the reaction mixtures were supplemented with 15% glycerol and 0.5% formamide for the S16 construct, with 3% DMSO for the N-terminal domain plus pET18a backbone (NBB) construct, and with 10% glycerol for the S8 inserts. All the PCR products were gel-purified.

The His-tagged N16C construct for *E. coli* expression was assembled in pET28a in two steps: first, the sequences encoding the 16-mer module (S16), as well as the N- and C-terminal domains were cloned into pET28a and served as PCR templates for the following Golden Gate assembly. In the second step, the N16C construct was assembled (Figure 2). For the S16 assembly (Step 1), two 8-mer (S8) repeat modules, obtained by PCR amplification using primers that introduce *BseR*I recognition and cut sites, and a linearized pET28a receiver vector containing the kanamycin resistance gene (Kan) and the N-terminal His-tag (50 fM each) were combined with 0.25 μL T4 DNA ligase and 0.25 μL *BseR*I in 1 μL 10× T4 DNA ligase buffer for a final reaction volume of 10 μL. The reaction was cycled 25 times for 2 min at 37 °C and 5 min at 16 °C, followed by 10 min at 60 °C, 10 min at 80 °C, and finally kept at 10 °C for final incubation.

In Steps 1b and 1c, the N- and C-terminal domains were assembled via *Bsa*I-based Golden Gate cloning by combining the linearized genes with the linearized pET28a vector. To promote protein purification of the individual terminal domains, a His-tag was added to the N- and C-termini of the N- and C-terminal domains, respectively. The same reaction conditions and thermocycling were used as for the S16-construct assembly, but the *BseR*I enzyme was replaced with *Bsa*I-HF.

For the N16C assembly (Step 2), the following three modules were combined: the His-tagged NTD together with the pET28a backbone (1), the linearized S16 module (2), and the CTD (3). The same *BseR*I-based Golden Gate assembly protocol was used as for the S16 construct.

The final Golden Gate products encoding for NTD, CTD, S16, and N16C were transformed into chemical ultracompetent XL10-Gold cells according to the manufacturer’s protocol and selected onto Luria–Bertani (LB) agar plates supplemented with 25 mg/L kanamycin and grown overnight at 37 °C. Selected clones were analyzed by restriction analysis, and their correct sequence was confirmed by Sanger sequencing.

### Protein Expression

For the expression of S16 and N16C, the respective expression constructs were transformed into the expression strains *E. coli* BL21(DE3). Single colonies were inoculated into the LB medium, supplemented with kanamycin antibiotics, and grown overnight at 37 °C. Ten ml of the overnight culture was transferred to 1000 mL of either LB or terrific broth (TB) medium and grown at 37 °C until an OD_600_ = 0.6–0.8 was reached. Protein expression was induced by the addition of 0.1 mM isopropyl-β-d-thiogalactopyranoside (IPTG), and cells were further grown at 37 °C, with 200 rpm shaking overnight. Cells were harvested by centrifugation at 7460*g*, 30 min, and 25 °C and stored at −80 °C for later protein purification.

To obtain ^13^C–^15^N-labeled N16C for the NMR studies, cells were grown in 2 L LB-Kan until an OD_600_ = 0.6 was reached. Cells were collected by centrifugation (7460*g*, 10 min, and 25 °C) and transferred into 0.5 L M9 minimal medium without carbon and nitrogen sources and incubated for 40 min at 37 °C. This was followed by the addition of 1 g of ^13^C-labeled d-glucose and 0.25 g of ^15^N-labeled ammonium chloride and incubation for an additional 35 min, prior to induction of protein expression with 0.1 mM IPTG and shaking overnight at 37 °C. The next day, the cells were harvested by centrifugation and stored at −80 °C until protein purification.

### Purification of S16

For the purification of S16, the pellet from 2 L expression culture was resuspended in 200 mL of buffer A2 (20 mM Tris–HCl, 500 mM NaCl, pH 7.5), supplemented with 8 M urea, and incubated with magnetic agitation at room temperature overnight. After adjusting the pH to 4.0 with acetic acid, the sample was further incubated for 2 h at room temperature. Following centrifugation (27,220*g*, 4 °C, and 30 min), the supernatant was precipitated by adding (NH_4_)_2_SO_4_ to a final concentration of 1.32 M and incubated with agitation for another 6 h. The mixture was centrifuged again, and the resulting supernatant was further precipitated with 2.80 M (NH_4_)_2_SO_4_. The precipitate was solubilized in buffer A2 containing 8 M urea and loaded into a 5 mL HisTrap (Cytiva) column preequilibrated with 10 column volumes (CV) of buffer A2. After washing the column with 10 CV buffer A2, the sample was eluted using a gradient up to 300 mM imidazole over 5 CV of buffer B2 (20 mM Tris–HCl, 500 mM NaCl, 300 mM imidazole, pH 7.5). The sample was then dialyzed against water overnight at 4 °C. During dialysis, S16 remained in solution, while impurities precipitated out. The protein was concentrated and then stored at −80 °C for further use. The expression yield was around 23 mg.

### Purification of N16C

N16C was purified from inclusion bodies. First, the cells were resuspended in 40 mL of buffer A3 (20 mM Tris–HCl, 80 mM NaCl, and pH 9) supplemented with DNaseI and one tablet of EDTA-free complete protease inhibitor. Next, the cells were lysed by sonication on ice for 20 min in pulsed bursts. The inclusion bodies were harvested by centrifugation (27,220*g*, 30 min, and 4 °C), and the supernatant was discarded. The pellet containing the inclusion bodies was dissolved in 35 mL buffer A3 supplemented with 4 M urea and heated to 80 °C for 20 min with agitation. The solubilized protein fraction was cleared by centrifugation (27,220*g*, 30 min, room temperature), and the supernatant was filtered using a 0.2 μm cellulose acetate filter. Due to the low solubility of N16C in low-salt conditions (see the Results section), anion-exchange chromatography was done followed by Ni-affinity chromatography. Therefore, the sample was first loaded onto a 5 mL HiTrap Q HP anion-exchange column (Cytiva) preequilibrated with 10 CV of buffer A3. After washing the column with 10 CV buffer A3, the protein was eluted by a linear gradient from 80 to 500 mM NaCl using buffer B3 (20 mM Tris–HCl, 500 mM NaCl, and pH 9) over 15 CV. After analysis by sodium dodecyl sulfate polyacrylamide gel electrophoresis (SDS-PAGE), the corresponding fractions were pooled and injected again into a 5 mL HisTrap Ni-affinity column preequilibrated with 10 CV of A4 buffer (20 mM Tris–HCl, 500 mM NaCl, 20 mM imidazole, pH 9). After washing the column with 10 CV of buffer A4, N16C was eluted by a linear gradient from 20 to 500 mM imidazole using buffer B4 (20 mM Tris–HCl, 500 mM NaCl, 500 mM imidazole, and pH 9) over 15 CV. The fractions containing the target protein were pooled and dialyzed against distilled water at 4 °C overnight. During dialysis, N16C precipitated out. The samples were lyophilized and stored at −80 °C. From 1 L culture, about 45 mg (LB-medium) and 75 mg (TB-medium) were obtained.

All chromatographic steps were performed at room temperature on an ÄKTA Pure Fast Pressure Liquid Chromatography (FPLC, GE Healthcare, Germany) system equipped with a UV detector (λ = 280 nm) using flow rates according to the column properties. The purity and structural integrity of the proteins were analyzed by SDS-PAGE and Western blotting.

### SDS Gel Electrophoresis

Thirty μL of protein solution from the expression extracts or from chromatographic fractions were mixed with 10 μL SDS loading buffer (Thermo Fisher Scientific). Samples were boiled at 100 °C for 5 min and centrifuged at 27,220*g* for 10 min. Ten μL of each sample and 2 μL of protein standard (Broad range, New England Biolabs P7719S) were loaded on precast gels (Novex 10–20% Tricine Protein Gels, 1.0 mm). The gels were run for 40 min at 160 V in running buffer (0.1 M Tris–HCl, 0.1 M Tricine, 0.1% SDS, and pH 8.25). Gels were stained with Coomassie Blue 250 (CBR-250) staining solution for at least 1 h and rinsed in water.

### Western Blot Analysis

After SDS-PAGE electrophoresis, the proteins were transferred onto a 0.45 μm poly(vinylidene fluoride) (PVDF) membrane with an omniBLOT Mini Transfer system (Cleaver scientific) at 160 V, 230 mA for 2 h at 4 °C. The membrane was blocked in Tween-Tris-buffered saline with 5% (w/v) nonfat dry milk for 1 h and then incubated with antihexahistidine monoclonal antibody (Thermo Fisher Scientific) in Tween-Tris-buffered saline at a ratio of 1:2000 overnight at 4 °C. Reactivity was analyzed using goat antimouse peroxidase-conjugated secondary antibody (Thermo Fisher Scientific) via chemiluminescence detection according to the manufacturer’s protocol.

### Mass Spectrometry of N16C

To further verify the identity of N16C, peptide-mass fingerprinting was done. Therefore, the protein was digested with trypsin (Thermo Fisher Scientific), and the peptides were initially loaded on an Acclaim PepMap 100 μ-precolumn cartridge (5 μm, 100 A, 300 μm ID × 5 mm, Thermo Fisher Scientific). Then, peptides were separated at 40 °C on a PicoTip emitter (noncoated, 15 cm, 75 μm ID, 8 μm tip, New Objective) that was packed in-house with Reprosil-Pur 120 C18-AQ (1.9 μm, 150 A, Dr. A. Maisch GmbH). Mass spectrometric analysis was performed on an Orbitrap Eclipse Tribrid Mass Spectrometer (Thermo Fisher Scientific) coupled to an UltiMate 3000 Nano-HPLC (Thermo Fisher Scientific) via an EASY-Spray source (Thermo Fisher Scientific) with or without a FAIMS interface (Thermo Fisher Scientific). Experimental details such as solvent gradients and spectrometer settings are available in the Supplementary Methods section. Peptides were searched against the in silico digested UniProt database for N16C protein, in which one unique peptide was required for protein identification. The false discovery rate (FDR) was determined using a decoy database and set to 1% as thresholds for both peptide-spectrum match and protein levels. Label-free quantification (LFQ) intensities were calculated for each sample.

### Film Casting

Film formation was initially optimized with the nonlabeled N16C protein, prior to the application of the protocol for the ^13^C,^15^N-labeled N16C. To achieve the highly concentrated protein solution for film casting, the lyophilized N16C protein was dissolved in formic acid to 20% (w/v) and then incubated on a rotary mixer overnight. 400 μL of solution was deposited into a Petri dish onto a 2.2 cm diameter round glass substrate. The solvent was evaporated in a fume hood at room temperature. The film was then washed with 100% isopropanol for 2 h, followed by washing with water overnight.

### Solution-State NMR Spectroscopy

For the solution-state NMR experiments, the uniformly ^13^C,^15^N-labeled N16C sample was dissolved either in DMSO-*d*_6_ or in 95% H_2_O/5% D_2_O and then sonicated for 30 min until most of the protein was dissolved. Sodium trimethylsilylpropanesulfonate (DSS) was added for internal referencing with a final concentration of 1 mM. After centrifugation at 17,000*g*, 500 μL of the sample was transferred to a 5 mm NMR tube and sonicated for an additional 3 h. The protein concentrations in DMSO-*d*_6_ and in H_2_O/D_2_O were about 58 and 12 μM, respectively.

All solution-state NMR experiments were performed at 37 °C on a Bruker Avance III spectrometer operating at a ^1^H Larmor frequency of 800 MHz (18.79 T) equipped with a triple resonance, cryogenically cooled HCN TCI probe. ^1^H, ^13^C, and ^15^N shifts were calibrated to DSS based on IUPAC recommendations. Triple-resonance assignment experiments (HNCA, HNCO, HNCACO, HNCACB, and HCCCONH) were performed using band-selective excitation short-transient (BEST) techniques and analyzed using Cara.^75^ 2D ^1^H–^13^C heteronuclear single quantum coherence (HSQC), 2D ^1^H–^15^N heteronuclear multiple quantum coherence (HMQC), and 3D ^1^H–^1^H–^13^C nuclear Overhauser enhancement spectroscopy (NOESY)-HSQC, ^1^H–^1^H–^15^N NOESY-HSQC experiments were also performed. These experiments were all measured with the NMRlib extension of Topspin.^76^

### Solid-State NMR Spectroscopy

For the solid-state NMR measurements, a ^13^C,^15^N-labeled N16C film was prepared the same way as the unlabeled film and then packed into a 1.3 mm rotor with some added DSS. The protein film was hydrated by soaking the opened rotor in H_2_O overnight at room temperature.

Solid-state MAS NMR measurements were performed on a Bruker Neo 700 MHz NMR spectrometer equipped with a 1.3 mm HCN MAS probe. The spinning frequency was set to 55.55 kHz, and the temperature was regulated to a ∼25 °C nominal temperature assessed based on the difference in the DSS and H_2_O chemical shifts. ^1^H, ^13^C, and ^15^N chemical shifts in all solid-state spectra were referenced to internal DSS signal. Typical 90° pulse lengths for ^1^H, ^13^C, and ^15^N were 1.5, 2.7, and 3.5 μs, respectively. All ^1^H–^13^C or ^1^H–^15^N 2D experiments were recorded in a ^1^H-detected fashion with a recycle delay of 1.0 s.

Direct excitation 1D ^13^C spectra were recorded with either 1 or 25 s recycle delay; the number of scans were 8192 or 2048, respectively. The ^13^C-detected 1D ^1^H–^13^C CP MAS spectrum was recorded with 2048 scans and a recycle delay of 1.2 s. The ^1^H–^13^C CP step had a 14 kHz tangential shaped ^1^H rf pulse and a rectangular 41 kHz rf pulse on ^13^C with a contact time of 500 μs, and carrier was placed at 52 ppm. The refocused insensitive nuclei enhanced by polarization transfer (INEPT) spectrum was recorded with 8192 scans and a recycle delay of 0.8 s. The INEPT delay was set to 802 μs. In all 1D ^13^C spectra, a 12.5 kHz XiX decoupling was applied during the acquisition.

In the ^1^H–^13^C CP-based 2D experiment (HCH), the spectral width of the indirect dimension was 27,777 Hz, with a maximum *t*_1_ evolution time of 4.6 ms over 256 increments, recorded with 128 scans; the ^13^C transmitter frequency was set to 50 ppm. The ^1^H–^13^C CP step had a 65 kHz tangential shaped ^1^H rf pulse and a rectangular 10 kHz rf pulse on ^13^C with a contact time of 1.25 ms.

In the ^1^H–^15^N CP-based 2D experiment (HNH) the spectral width of the indirect dimension was 13,888 Hz, with a maximum *t*_1_ evolution time of 9.2 ms over 256 increments, recorded with 128 scans with the ^15^N transmitter frequency set to 120 ppm. During the ^1^H–^15^N CP, the ^1^H field strength was ramped with a tangential shape with an effective strength of 14 kHz and a 42 kHz rectangular shape pulse was applied on the ^15^N channel with a contact time of 150 μs. In the HNH and HCH experiments, a 12.5 kHz XiX ^1^H-decoupling was applied during the ^13^C or ^15^N acquisition, and a 10 kHz WALTZ-16 decoupling was used during ^1^H detection. The water signal was suppressed using the MISSISSIPPI scheme with 15 kHz irradiation for 80 ms.

In the ^1^H–^13^C INEPT-based 2D HSQC experiment, the spectral width of the indirect dimension was 27,777 Hz, with a maximum *t*_1_ evolution time of 10.8 ms over 600 increments, recorded with 64 scans with the ^13^C carrier placed to 60 ppm. The INEPT delay (1/4*J*) was set to 2 ms.

In the ^1^H–^15^N INEPT-based 2D HSQC experiment, the spectral width of the indirect dimension was 13,888 Hz, with a maximum *t*_1_ evolution time of 10.8 ms over 300 increments, recorded with 64 scans with ^15^N transmitter frequency set to 120 ppm. The INEPT delay was set to 2.17 ms.

Homonuclear ^13^C–^13^C mixing was achieved with dipolar recoupling enhanced by amplitude modulation (DREAM) scheme with a mixing time of 4 ms, and a tangential shaped pulse centered at 56 ppm.^77^ In the DREAM experiment, the spectral width of the indirect dimension was 55,555 Hz, with a maximum *t*_1_ evolution time of 5.4 ms over 600 increments, recorded with 128 scans.

For both CP-based and HSQC-based ^13^C *R*_1ρ_ experiments, two sets of on-resonance measurements were recorded where the carrier was placed either to 20 ppm or to 53 ppm. The spin-lock field strength on-resonance was set to 7.5 kHz, and spin-lock pulse lengths were 5, 10, 15, 20, 25, and 30 ms. The CP-based and HSQC-based ^13^C *R*_1_ experiments were recorded with a carrier placed at 40 ppm. The relaxation delay was set to 0.2, 0.5, 0.75, 1, 2, 3, 4, 5, 6, 7, 8, 9, and 10 s in the CP-based and to 20, 50, 80, 100, 150, 200, 400, and 500 ms in the HSQC-based experiments. In all relaxation measurements, the spectral width of the indirect dimension was 13,888 Hz with a maximum *t*_1_ evolution time of 3.6 ms (CP) or 7.2 ms (HSQC) over 100 or 200 increments recorded with 64 scans. The ^1^H–^13^C CP step had a 70 kHz tangential shaped ^1^H rf pulse and a rectangular 15 kHz rf pulse on ^13^C with a contact time of 500 μs. The recycle delay was 0.8 s for the *R*_1_ and 1 s for the *R*_1ρ_ measurements. The CP-based and HSQC-based on-resonance ^13^C *R*_1ρ_ are standard proton-detected HCH or refocused HSQC correlation spectra with an inserted relaxation period, where the ^13^C_*y*_ coherence is spin-locked for a period of time *t*_rel_ with adiabatic spin-lock and with a decoupling π pulse on the proton channel in the middle of the spin-lock period. The CP-based and HSQC-based ^13^C *R*_1_ experiments are a variation of the ^13^C *R*_1ρ_ pulse schemes without the spin-lock on the ^13^C channel. The applied pulse schemes are shown in Figures S3 and S4.

*R*_1ρ_ rate constants were obtained by fitting the peak intensities in the series of 2D HC correlation spectra with variable time delays with monoexponential decays. For *R*_1_ rate constants, the peak intensity decays were fit with both mono–biexponential functions, and a statistical *F*-test was performed to decide which function gives a better fit. Fitted and derived relaxation parameters are listed in Tables S4–S6.

## Results

### Protein Design, Expression, and Purification

We designed an artificial spider silk protein consisting of the NTD based on the amino acid sequence of the NTD of *Nephila clavipes* MaSp2, a tandem repeat unit with 16× repetition, and a CTD derived from the *N. clavipes* MiSp CTD. The terminal domains were selected from the extensive list of spider silk terminal domains collected by Hayashi et al.^78^ based on their predicted high solubility and stability. Additionally, we chose terminal domains that did not contain any cysteines to avoid disulfide-bond formation between two monomers, resulting in aggregation during protein expression or purification. The amino acid sequence of the core domain was based on the consensus sequence of MaSp1 dragline silk of *N. clavipes* with some further modifications, e.g., a “GPGGX” motif was placed into the Gly-rich segment, which is characteristic of MaSp2 sequences and is known to enhance the elasticity of the silk fibers by facilitating β-spiral formation;^79^ moreover, a “GPA” amino acid triplet was also introduced at the junction of the Gly-rich and Ala-rich segments. The “GPA” triplet at the interface between the amorphous and crystalline domains of the designed tandem repeat proteins based on the sequence of squid ring teeth proteins was found to be critical for the exceptional thermal conductivity of the produced biomaterial.^80^ The amino acid sequence shown in Figure 1 was encoded as a synthetic gene (N16C) with the sequence optimized for genetic stability and the heterologous expression in *E. coli*. To facilitate protein purification by Ni-affinity chromatography and enable direct identification of the protein by Western blotting using anti-His-tag antibodies, the designed recombinant spider silk protein sequence was fused with an N-terminal hexa-histidine tag (His).

The seamless cloning of tandem repeat proteins poses substantial challenges, such as primer design and plasmid instability due to recombination events. Therefore, to generate the expression constructs for the three separate domains (NTD, CTD, and S16) as well as the full-length construct (N16C), the Golden Gate assembly was used. Golden Gate assembly has the advantage of being extremely modular, allowing for complete flexibility in protein composition, length, and architecture.^60,81,82^ Due to the nature of type IIs endonucleases used here, the restriction sites are removed during the cloning process, allowing for repeated cycles of digestion and ligation, leading to the accumulation of the final, desired construct. Moreover, Golden Gate assembly is a seamless cloning method as it does not leave any extra nucleobases at the restriction enzyme cleavage sites that would disrupt the amino acid sequence of the translated genes that have been assembled.

First, we generated expression constructs for the three separate domains: NTD, CTD, and the core domain, containing 16 repeats (S16), which were then used to assemble the expression construct consisting of the NTD, S16, and the CTD (N16C). In order to avoid primer binding to multiple sites in the repetitive sequences, the primers were designed to be complementary to a unique 8-bp-long sequence directly before and after the repeat module and contained the *BseR*I type II restriction endonuclease site, which cuts eight base pairs away from the binding site and leaves a 2-bp-long overhang. Moreover, to facilitate directional cloning, we introduced different but complementary cleavage site sequences and performed restriction and ligation in a one-pot reaction. With this strategy, we successfully generated the desired expression constructs encoding engineered synthetic spider silk genes that mimic the sequence and architecture of natural silk (Figure 2).

**Figure 3:**
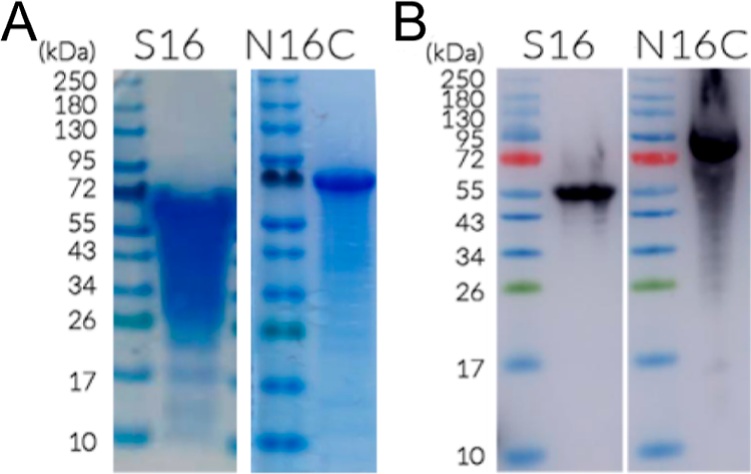
Purified S16 and N16C proteins were analyzed by SDS-PAGE followed by (A) Coomassie brilliant blue staining and (B) Western blot with anti-His antibody.

Expression and purification of the synthetic S16 and N16C were optimized, regarding expression strain, used media, expression time, and temperature. Both S16 and N16C were expressed insolubly in *E. coli* and were therefore purified from inclusion bodies. Spider silk protein films are commonly formed from protein dissolved in water, hexafluoro-2-propanol (HFIP), formic acid, or trifluoroacetic acid, with salts like NaCl interfering with film casting.^83,84^ Therefore, S16 and N16C were dialyzed against water, where N16C precipitated out while S16 remained in solution. Figure 3 shows the SDS-PAGE and Western blot analysis of the purified S16 and N16C. Here, they run at a higher molecular weight than expected (at about 65/80 kDa instead of 43/68 kDa for S16/N16C). It is known that proteins deficient in bulky residues (e.g., antifreeze proteins, collagen) migrate anomalously on gels and stain poorly with Coomassie blue dye.^85^ For N16C, we were able to obtain overall yields in the range of 45–75 mg pure protein per liter *E. coli* culture, depending on the expression conditions. Similar yields have been reported for other spidroin mimics.^42,86−88^ Expression yield of S16 was markedly lower, in the range of 15–23 mg/L.

In order to obtain ^13^C–^15^N-labeled N16C for the NMR characterization, protein expression was done in M9 minimal medium with ^13^C-labeled d-glucose and ^15^N-labeled ammonium chloride as sole sources for carbon and nitrogen, respectively. The purity and identity of the expressed proteins were confirmed by SDS-PAGE, followed by Western blotting analysis using a monoclonal antibody against the His-tag (Figure 3). The identity of the purified N16C was further confirmed by tryptic digestion and peptide mass-fingerprinting (Table S2).

The lyophilized N16C powder was largely insoluble in aqueous solvents or HFIP but readily dissolved in formic acid. The freshly prepared N16C solution was transparent, but overnight it turned pink, and in 3 weeks at room temperature, the color changed gradually to blue and further to black (Figure S1). The color change indicates an unstable colloidal protein solution with increasing particle size. In contrast, S16 dissolved in water, HFIP, and formic acid, and the solution did not display any color change in any of these solvents, implying that in the absence of the terminal domains, the repeat protein does not self-assemble.

The freshly prepared N16C film cast from formic acid solution was insoluble in water, unlike the film prepared from S16 (Figure S2). This implies that even in the strongly denaturing formic acid solution, the terminal domains keep the repeat units in such a preorganized conformation where the gradual solvent evaporation enhances the intermolecular interactions and facilitates the ordered dynamic protein self-assembly into a chemically stable material. As-cast dry N16C films were brittle but became elastic when plasticized with isopropanol and water. As long as the films were hydrated, they were soft and elastic, and they became brittle again when water evaporated.

### Solution-State NMR Analysis of N16C

**Figure 4:**
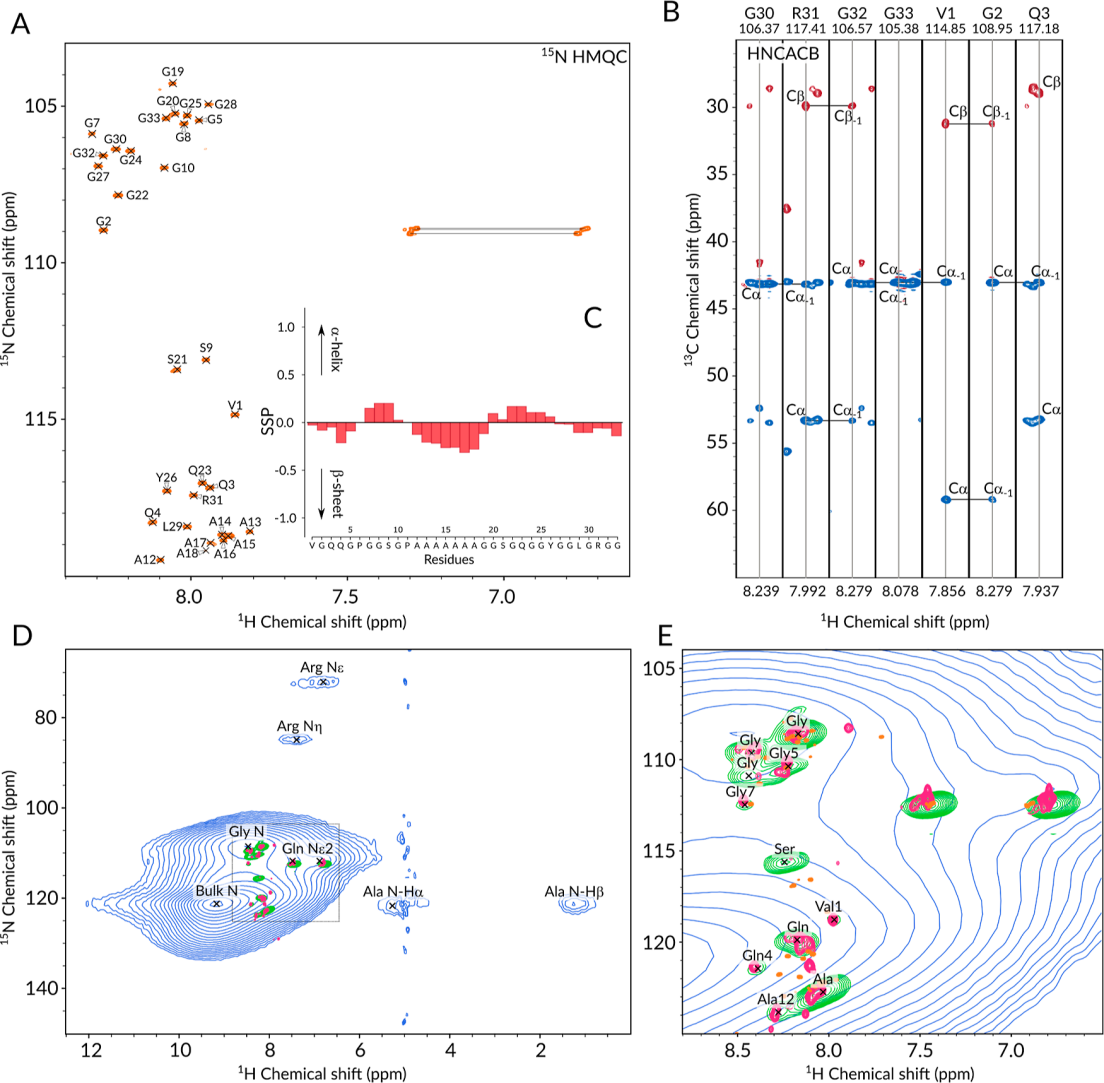
(A) ^1^H–^15^N HMQC spectrum of ^13^C,^15^N-labeled N16C in DMSO-*d*_6_ measured at 800 MHz ^1^H Larmor frequency at 37 °C. (B) Representative strips from the 3D HNCACB spectrum used for backbone and side-chain carbon assignment. (C) SSP plot calculated with ncSPC^89^ employing ^13^Cα, ^13^Cβ, ^1^Hα, and amide ^15^N chemical shifts. (D) ^1^H–^15^N CP-based HNH (blue) and INEPT-based HSQC (green) correlation spectra of ^13^C^15^N-labeled hydrated N16C film measured at 700 MHz ^1^H Larmor frequency at 55.55 kHz MAS frequency overlaid with the ^1^H–^15^N HMQC spectrum of ^15^N-labeled N16C in water measured at 800 MHz ^1^H Larmor frequency at 25 °C (magenta). (E) Zoomed in illustration of D overlaid with the solution-state ^1^H–^15^N HMQC spectrum of N16C recorded in DMSO-*d*_6_ (orange). Partial assignment of the solution-state HMQC spectrum in water was transferred to the solid-state spectrum.

To verify the amino acid connectivity and to elucidate the solution-state structure of the protein, we performed double- and triple-resonance NMR assignment experiments on N16C dissolved in DMSO-*d*_6_. Attempts to analyze the spectra of N16C dissolved in 95% H_2_O/5% D_2_O resulted only in the incomplete assignment of the core repetitive unit (Figure 4E, magenta spectrum). Broad resonances, a greater number of C–H signals with varying peak intensities, and weak coherence transfer in 3D experiments hint at a slowly tumbling, structurally heterogeneous molecule in aqueous environments. The observed ^1^H^*N*^ and ^15^N^*H*^ chemical shifts for Ala, Gln, and Gly residues resemble the chemical shifts of the native spider silk protein collected from the major ampullate gland of *Latrodectus hesperus,* which was found to be highly flexible and unstructured.^90^ Contrarily, the sharp signals and excellent magnetization transfer in multidimensional experiments of the N16C sample measured in DMSO-*d*_6_ allowed for the complete assignment of the 33-amino-acid-long repetitive unit (Figure 4A). Due to the 16-fold higher signal-to-noise ratio of the repetitive core with respect to the nonrepetitive terminal domains, the detection and subsequent assignment of the terminal units could not be achieved. The narrow ^1^H^*N*^ chemical shift distribution as well as the sharp resonances suggest that N16C in DMSO solution appears as an intrinsically disordered protein. The disordered nature was further confirmed by the secondary structure propensity (SSP) analysis that uses the neighbor corrected ^1^Hα, ^13^Cα, ^13^Cβ, and ^15^N chemical shifts to report on the likelihood of α-helical or β-strand backbone conformations (Figure 4C). In the disordered polypeptide, only the poly(A) stretch stands out with a slight tendency to form extended β-strands.

### Structural Insights of N16C Film

The functionally relevant state of both natural and bioengineered silk proteins is their solid state, e.g., when they are spun into fibers or assembled into non-natural morphologies, such as into films, hydrogels, spheres, or foams. In our study, we prepared a ^13^C, ^15^N-labeled N16C film and measured its solid-state MAS NMR spectra at a spinning frequency of 55.55 kHz at 700 MHz ^1^H Larmor frequency in a 1.3 mm HCN probe. The film was washed with H_2_O and kept hydrated before loading into the rotor. Due to the heterogeneous hydrogen-bonding environment of the semicrystalline protein film, the amide ^1^H and ^15^N resonances show a large distribution, which leads to extremely broad overlapping peaks in cross-polarization (CP)-based experiments (see Figure 4D displaying the CP-based ^1^H–^15^N correlation spectrum in blue). Thus, besides a few side-chain resonances, only the glycine (8.5, 108.5 ppm) backbone signals can be distinguished from the rest of the bulk amide backbone resonances. Ala ^15^N^*H*^ (121.4 ppm) was assigned to be part of the large bulk signal based on its cross-peak with the Ala Hα (5.22 ppm) and Ala Hβ (1.38 ppm) resonances. The downfield-shifted ^1^H^*N*^ resonance of Ala together with the bulk (9.25 ppm) suggests a strongly hydrogen-bonded solid material indicative of interstrand molecular contacts as it is expected in a β-nanocrystal.^63^

**Figure 5:**
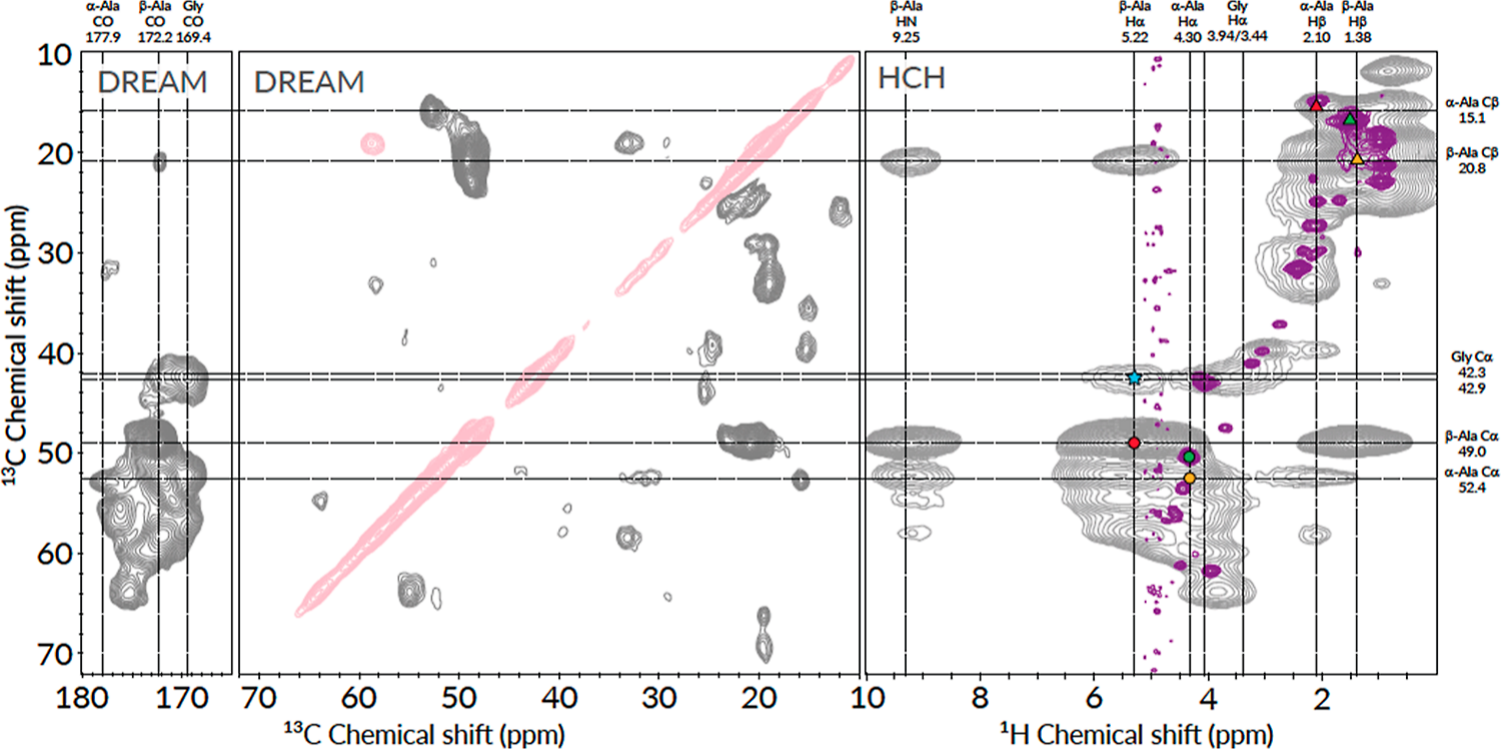
^13^C–^13^C and ^1^H–^13^C 2D correlation spectra of ^13^C^15^N-labeled hydrated N16C film measured at 700 MHz ^1^H Larmor frequency at 55.55 kHz MAS frequency. Left and middle panel: ^13^C–^13^C DREAM, right panel: CP-based ^1^H–^13^C HCH spectrum (gray) overlaid with the INEPT-based solid-state ^1^H–^13^C HSQC spectrum (purple). Dashed lines indicate the chemical shifts of the alanine and glycine resonances; α-Ala and β-Ala terms stand for alanines in α-helical and β-sheet conformations, respectively. Red, green, and yellow circles or triangles mark the positions of Hα-Cα or Hβ-Cβ cross-peaks that belong to the rigid β-sheet, rigid α-helix, and to the solubilized random coil conformations of alanine. Blue star labels the Gly Cα-Ala Hα cross-peak. The assigned ^1^H–^13^C HSQC and HCH spectra are shown in Figures S5 and S6.

The broad signals observed in the CP-based experiments stem from immobile residues that constitute the rigid core of the cast film. To inspect the effect of hydration on the structure and dynamics of the film, we also recorded INEPT-transfer-based HSQC experiments, where only those resonances emerge that have long coherence lifetimes due to motion-induced dipolar decoupling.^91^Figure 4D,E displays the overlay of the solid-state CP-based (blue), the solid-state INEPT-based (green), and two solution-state HMQC ^1^H–^15^N correlation spectra, one recorded in H_2_O (magenta) and the other one in DMSO-*d*_6_ (orange), and Figure 5 shows the corresponding overlay of the ^1^H–^13^C CP-based (HCH in gray) and INEPT-based (HSQC in purple) spectra (the overlay of the solid-state and solution-state ^1^H–^13^C HSQC and HMQC spectra is displayed in Figure S5). Clearly, the INEPT-based aqueous solution- and solid-state spectra match each other, indicating that there is some solubilized, highly flexible N16C present in the hydrated film sample. The partial assignment of the solid-state HSQC spectrum in Figure 4E stems from the tentative assignment of the solution-state H_2_O/D_2_O spectrum. When comparing the INEPT and CP-based solid-state spectra of N16C, it is noticeable that the Gly ^1^H–^15^N (and ^1^H–^13^C) resonance frequencies are relatively unchanged, while the Ala ^1^H–^15^N (and ^1^H–^13^C) resonances shift remarkably downfield in ^1^H (from 7.9–8.4 ppm to 9.2 ppm) and upfield in ^15^N (122.8 to 121.4 ppm) dimensions as the protein transitions from the soluble to its insoluble state. This divergence implies that the hard segments of the repetitive units of N16C go through a major structural rearrangement when the water-solubilized form assembles into a solid film, while the structure of the glycine-rich soft segments remains similar in the solubilized and solidified states.

To confirm that the poly(A) region in the core domain of N16C film has the expected extended conformation, we assigned the Ala chemical shifts in CP and INEPT-based ^1^H–^13^C and CP-based ^13^C–^13^C correlation (DREAM) spectra (Figure 5 and Table 1). Ala CO, Cα/Hα, and Cβ/Hβ resonances are excellent indicators of the local backbone conformations, therefore we could readily assign the peaks that belong to Ala in α-helical or β-strand conformations. The majority of the Ala resonances appeared at positions characteristic of β-strand (CO: 172.2 ppm, Cα: 49.0 ppm, Cβ: 20.8 ppm), and a smaller fraction of peaks appeared at α-helical positions (CO: 177.9 ppm, Cα: 52.4 ppm, Cβ: 15.1 ppm). Note that the peak intensity ratios in CP-based experiments reflect not only the population ratios but also the magnetization-transfer efficiencies, where the rigid segments appear with apparently stronger intensities due to more efficient magnetization transfer. Therefore, the peak-intensity comparison gives only qualitative insights into the structural preference of the poly(A) segments. The presence of both helical and β-sheet conformations for Ala (and also for Gly) has been observed in the water-plasticized state of native *N. clavipes* and *L. hesperus* spider silk fibers, however, were assigned to belong to sequentially different Ala, namely, to the ones in poly(A) and in GGA sequences, the latter of which is part of the Gly-rich amorphous segment.^20,25^ In our model system, Ala exists only in the context of the poly(A) segments, thus the observed distinct chemical shifts reflect the structural heterogeneity of the hard core.

**Table 1:** ^13^C and ^1^H Chemical Shift of N16C Film Compared to Literature Values for Random Coil, β-Strand, and α-Helix Chemical Shifts^a^^,^^92^

	experimental shifts					dragline silks	
residue	CP major/minor	HSQC	coil	β-sheet	α-helix	fiber^22,25,31,63,65^	gland^64,90^
Ala CO	172.2/177.9		177.7	176.1	179.4	174.5/174.6/176.1	178.0
Ala Cα	49.0/52.4	50.4	52.8	51.5	54.8	51.0/51.2/52.0/50.3	52.6/53.0
Ala Cβ	20.8/15.1	16.8	19.1	21.1	18.3	19.4/22.9/25.3/22.1	19.1/19.2
Ala Hα	5.22/4.30	4.3	4.26	4.77	4.03	5.1/4.2	4.23/4.30
Ala Hβ	1.38/2.10	1.46	1.36	1.36	1.36		1.38
Ala HN	9.25	8.05–8.27	8.15	8.44	8.08	8.2/9.0	8.1/8.25
Gly CO	169.4/171.7		173.9	172.6	175.5	173.0	171.8/174.3/176.5
Gly Cα	42.9/42.3	43.1	45.5	45.2	46.9	45.3/43.0/44.7	44.7/45.5
Gly Hα	3.94/3.44	4.06	3.96	4.20	3.81	2.9/4.8	3.95
Gly HN	8.5	8.15–8.46	8.33	8.34	8.29	8.2/9.0	8.19–8.40
Gln CO	172.2		175.9	174.9	178.0	173.8/174.5	171.8
Gln Cα	52.4	53.6	56.1	54.8	58.5	54.6/54.1	56.0/56.1/56.5
Gln Cβ	31.4	30.2	29.1	31.3	28.5	33.7/29.1	29.8/29.5
Gln Hα	5.57	4.44	4.26	4.80	3.99		4.35
Gln HN	9.22	8.39/8.19	8.23	8.48	8.04		8.26/8.35
Pro CO	173.7		176.9	176.2	178.3	176.8/175.5	177.7
Pro Cα	60.3	61.8	63.5	62.6	65.5	62.7/62.1	63.6
Pro Cβ	30.3	27.3/29.9	31.9	32.3	31.5	32.5/31.3	32.1
Pro Cγ	25.2	24.9	27.3	27.3	27.3	27.4/26.6	27.2
Pro Cδ	47.5	47.5	50.5	50.5	50.5	49.5/49.0	49.8
Pro Hα	4.28	3.96	4.37	4.60	4.22		
Ser CO	175.1		174.5	173.6	175.9		174.7
Ser Cα	55.0	56.1	58.4	57.5	60.9	57.2/56.3	58.7/58.6
Ser Cβ	63.8		64.0	65.2	63.1	63.3/65.7	64.0/63.9
Ser Hα	5.54	4.57	4.47	4.91	4.25		
Tyr CO	174.4		175.4	174.5	177.4	174.2/174.5	
Tyr Cα			58.0	56.8	61.0	57.0/58.0	
Tyr Cβ	37.2	37.2	39.0	41.0	38.3	40.3/39.4	
Tyr Cγ	129.1		130.9	130.9	130.9	129.0/129.2	
Tyr Cδ	129.1	130.9	132.9	132.9	132.9	132.0/132.3	
Tyr Cδ	115.9	115.9	118.1	118.1	118.1	115.7/117.4	
Tyr Cδ	157.2		157.0	157.0	157.0	157.2	
Val CO	174.4		175.7	174.8	177.7		
Val Cα	58.5	61.2	62.1	60.8	66.2		
Val Cβ	33.0	31.5	32.7	33.9	31.5		
Val Cγ	19.2	18.9	21.6	21.6	21.6		
Val Hα	5.03	4.49	4.12	4.60	3.58		
Leu CO	176.2		176.9	175.7	178.5		
Leu Cα	55.3		54.9	54.1	57.5	55.4	
Leu Cβ	39.8	39.9	42.4	43.8	41.6		
Leu Cγ	24.9	24.8	26.9	26.9	26.9		
Leu Cδ1	23.7	22.9	24.9	24.9	24.9		
Leu Cδ2	23.1	21.2	24.2	24.2	24.2		
Leu Hα	4.40		4.36	4.82	4.00		

a Reference values are also given for dragline silk of *N. clavipes*(22,61,64,65,90) and *L. hesperus*.^25^ For all Pro entries, values for the trans isomer are reported. All shifts are in ppm, referenced to DSS, and TMS → DSS conversion was achieved via the addition of 1.7 ppm.

Besides the two solid-phase Ala environments that are visible only in CP-based experiments, we also observed a third Ala conformation that belongs to the mobile segments appearing primarily in the HSQC spectra. Based on its Cα: 50.4 ppm and Cβ: 16.8 ppm chemical shifts, this solubilized Ala is likely part of a loop or β turn-like structure,^19^ while the ^1^H^*N*^: 8.0–8.3 ppm and ^15^N: 123.3 ppm suggest a random coil conformation. In the solid-state ^1^H–^13^C HSQC spectrum, not only the fully solubilized Ala signals emerge but also the Hβ/Cβ peak of the α-helical Ala (red triangle in Figure 5), suggesting that the α-helical segments are in direct contact with the surrounding water and their methyl side-chains protrude into the solvent, while their backbones are still part of the rigid core (the Hα/Cα peak of the α-helical Ala did not appear in the HSQC spectrum).

Previous reports on native dragline silks suggested that the secondary structure of Gly in the soft segments forms disordered 3_1_-helices^24,25^ or elastin-like type II β-turns (in the GPGXX units in MaSp2 proteins),^19^ while a smaller fraction of Gly that flanks the poly(A) segments is part of the β-nanocrystals and found in an extended β-sheet conformation.^19,25,31^ In our hydrated film sample, we observed two Gly environments in the CP-based spectra, where the resonance frequencies of the two states differ both in their chemical shifts and line widths. A smaller fraction with heterogeneously broadened peaks (^13^Cα line width 3.3 ppm, ^1^Hα line width: 0.8 ppm) appears at CO: 171.7 ppm, Cα: 42.3 ppm, Hα: 3.44 ppm and a larger fraction with narrower line width (^13^Cα line width: 1.6 ppm, ^1^Hα line width 0.7 ppm) at CO: 169.4 ppm, Cα: 42.9 ppm, Hα: 3.94 ppm. The former set of resonances resembles the values of a random coil or the shifts of 3_1_-helices, while the latter set of shifts is more indicative of an extended conformation. In the ^1^H–^13^C HSQC spectrum (Figure 5 purple spectrum), the Gly Cα/Hα peak appears close to the resonance frequency of the major Gly Cα/Hα of the CP-based HCH spectrum, suggesting a chemical environment that is similar in both solubilized and solidified film parts. In the HCH spectrum, a strong cross-peak between the Gly Cα and Ala Hα in β-sheet (blue star in Figure 5) reveals spatial proximity between Gly in extended conformation and Ala in β-sheets. The intensity of the cross-peak suggests that it is not just a sequential cross-peak (there is only one occasion of an Ala–Gly neighbor in the repeat sequence) but rather indicates that a substantial part of the Gly-rich segment is incorporated in the β-nanocrystals.

Besides the Ala and Gly resonances, we tentatively assigned the ^13^C and ^1^Hα resonances of most other amino acids in the repetitive core based on their chemical shifts and expected CC connectivities (Figure 5). These resonances along with the typical α-helical, β-strand, and random coil conformations^92^ as well as the chemical shifts of the silk protein from the *N. clavipes*, *A. aurantia*, and *L. hesperus* major ampullate gland,^64,90^ and dragline fiber^19,22,31,63,65^ are tabulated in Table 1. Note that many peaks of the repetitive core were broadened beyond detection in the ^1^H–^13^C HSQC spectrum either due to ms−μs time-scale exchange processes or residual anisotropic interactions. The affected amino acids include the Hα/Cα and/or Hβ/Cβ resonances of Tyr, Ser, Val, Leu, and Arg. The observed chemical shifts in the HSQC spectrum are between the values of the solid shifts and the shifts of the dragline silk proteins of the major ampullate gland of *N. clavipes,* indicating that regardless of their high mobility, the solubilized segments retain some of their solid-state secondary structure.

### Solid-State Dynamics of N16C Film

**Figure 6:**
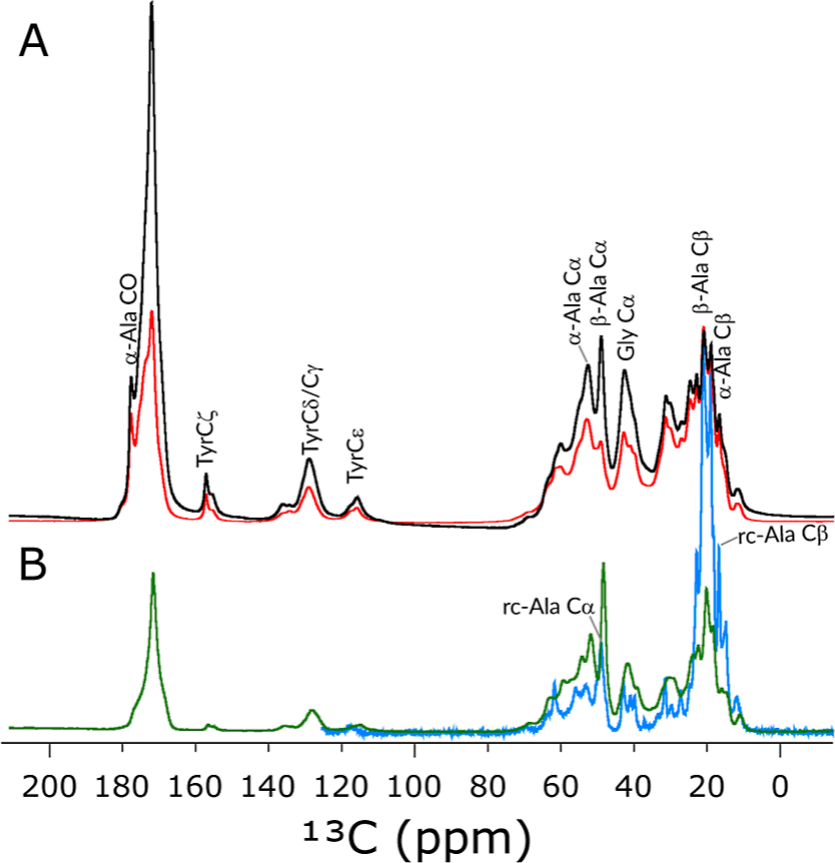
(A) Overlay of two direct-excitation ^13^C spectra of N16C film recorded with 1 s (red) or 25 s (black) recycle delay at 700 MHz ^1^H Larmor frequency at 55.55 kHz MAS. The spectra are scaled to the same number of scans. The relative increase in peak intensity in the spectrum with short delay indicates mobile residues. (B) Overlay of the ^1^H–^13^C CP MAS spectrum (green) with the r-INEPT spectrum (blue). The ^1^H–^13^C CP used a double-quantum CP condition with a 500 μs contact time. Assignments of selected peaks are displayed in the figure.

We gained the first insight into the hydration-induced chain dynamics of N16C film by comparing four 1D ^13^C spectra (Figure 6). These included two spectra recorded with direct ^13^C excitation (DD) with short (1 s) and long (25 s) recycle delay and two spectra with ^1^H excitation followed by either ^1^H–^13^C CP or refocused INEPT (r-INEPT) transfer steps. Figure 6A shows the overlay of the two DD spectra, while Figure 6B compares the CP MAS and r-INEPT spectra. In the DD spectra, the relative increase in peak intensity in the spectrum with short delay indicates residues with increased mobility on the ns time scale. In CP MAS spectra, the signal intensities of the immobile nuclei are relatively enhanced with respect to the ones with fast (ns) or intermediate (μs) time-scale of motion due to strong anisotropic dipolar coupling. In contrast, the r-INEPT spectrum selectively displays the mobile segments with fast isotropic motion.

As expected, the methyl region has the highest mobility on all time scales as the signals around 20 ppm (including Ala Cβ, Val Cγ, and Leu Cδ signals) are relatively enhanced both in the r-INEPT as well as in the DD spectrum recorded with a 1 s delay (Figure 6 blue and red curves). It is more interesting to compare the signals in the Ala Cα region (∼50 ppm), where the Ala Cα signal of the β-sheet conformation is relatively enhanced in the CP spectrum, while it is reduced in the DD spectrum recorded with a 1 s delay. In contrast, the opposite is true for the Ala Cα signal of the α-helical conformation. A third Ala Cα signal with random coil chemical shift appears in the r-INEPT spectrum, while the Cα shifts of the ordered helical and extended conformations are not observable here.

The low resolution of the 1D spectra of the uniformly ^13^C-labeled sample hinders any in-depth dynamic analysis. Therefore, to further investigate the role of water in the internal motion of the soft and hard segments of the film made from our dragline-silk-mimicking protein, we recorded ^13^C *R*_1_ and *R*_1ρ_ relaxation experiments based on 2D ^1^H–^13^C correlation spectra. The pulse sequences were tailored such that we could selectively report on the mobility of the rigid segments using CP-based experiments as well as on the solvent-exposed flexible segments employing INEPT transfers in the pulse sequence (see the Supporting Information for details on the pulse sequence setup).

Since N16C is fully protonated and uniformly ^13^C-labeled, the derived relaxation rate constants are substantially affected by ^1^H–^1^H and ^13^C–^13^C spin diffusion as well as unsuppressed coherent contributions. Accordingly, the relaxation profiles are multiexponential, and the fitted rates can provide only qualitative insights into the spin dynamics. Nevertheless, we measured and compared the *R*_1_ and *R*_1ρ_ decay profiles in a pairwise fashion always comparing the same resonances to each other, e.g., we contrast the Ala Cα rates in the CP- and HSQC-based experiments. *R*_1_ rates were fit with both mono- and biexponential functions, and an *F*-test was used to identify the best fit. Generally, the decays in the CP- and HSQC-based *R*_1_ experiments were biexponential and monoexponential, respectively. *R*_1ρ_ decays were only fit with monoexponential functions. To aid the residue-specific comparison of the *R*_1ρ_ rates, we give the offset-compensated rate constants as *R*_1ρ_/sin^2^ β, where β is the offset angle.

**Figure 7:**
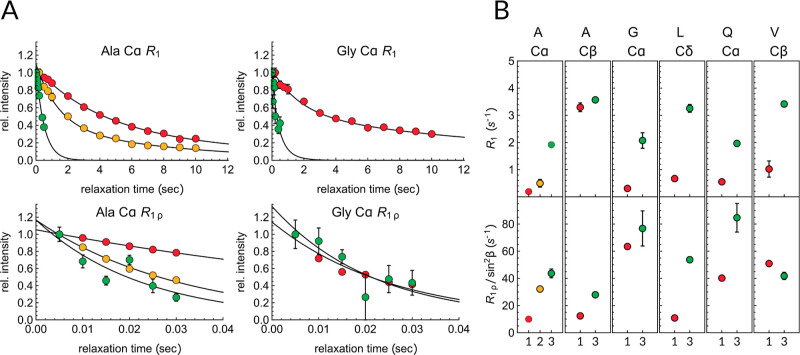
(A) Representative *R*_1_ (upper row) and *R*_1ρ_ (bottom row) relaxation decays of Ala Cα and Gly Cα resonances. Red, yellow, and green symbols represent the sites that belong to the extended, helical, and random coil conformations, respectively. Solid lines are the best monoexponential (*R*_1ρ_) or biexponential (*R*_1_) fits of the decays. (B) Representative *R*_1_ (upper row) and *R*_1ρ_/sin^2^ β (bottom row) relaxation rate constants of Cα, Cβ, or Cδ sites of N16C. Red (1), yellow (2), and green (3) symbols represent the sites that were observed in the CP-based (1 and 2) and in the HSQC-based (3) experiments. For Ala Cα, the rates were determined for the extended (red, 1), α-helical (yellow, 2), and random coil (green, 3) conformations. The decays and strips of all other sites are shown in Figures S7–S9.

Substantial signal intensity loss in the relaxation experiments allowed the observation of only a handful of peaks in both CP-based and HSQC-based relaxation experiments. Figure 7A shows the *R*_1_ and *R*_1ρ_ decay profiles of Ala Cα and Gly Cα sites as they were observed in the CP-based (red and yellow) or in the HSQC-based (green) experiments; all other decay profiles are displayed in Figures S7 and S8. For Gly in the CP-based spectra, only the major Cα peak was detectable, and hence, its rates report on the Gly-rich segment that is incorporated into the β-nanocrystals.

*R*_1_ and *R*_1ρ_ relaxation rate constants are most sensitive to motions on the ns and μs time scales, respectively, where higher rate constants generally indicate increased mobility. As expected, both the *R*_1_ and *R*_1ρ_ of almost all ^13^C signals in the HSQC-based experiment were markedly higher than the corresponding rate constants in the CP-based experiment (Figure 7), e.g., the major Ala Cα *R*_1_ increased from 0.16 ± 0.001 to 1.92 ± 0.05 s^–1^, and Ala Cα *R*_1ρ_ increased from 9.9 ± 0.05 to 43.5 ± 6.4 s^–1^. In the CP-based relaxation measurements, only the Cα resonance of the α-helical Ala was observable, while the Cβ resonance broadened beyond detection; its *R*_1_ and *R*_1ρ_ values of 0.28 ± 0.01 and 32.2 ± 0.9 s^–1^, respectively, are in between the values of the β-stranded Ala Cα and the solubilized Ala Cα. The higher mobility of the helical Ala with respect to the β-stranded Ala likely stems from solvent exposure and from the reduced packing in helical conformations.

To report on the dynamics of the Gly-rich segment of the N16C film, we compared the relaxation of Gly Cα, Gln *C*α, Val Cβ, and Leu Cδ resonances in the solid and solubilized forms (Figure 7). In their solid state, their ns-time scale dynamics resemble the dynamics of the hard segments with *R*_1_ rates in the range of 0.3–1.0 s^–1^ (which is still two to five times higher than the *R*_1_ of Ala Cα). Likewise, they gain much ns-time scale mobility when they are directly solubilized by water apparent from the elevated overall *R*_1_ rates of the signals in the HSQC-based spectrum. Interestingly, the observed solid-state Gly Cα *R*_1_ rate is among the lowest ones (0.31 ± 0.10 s^–1^). Taking into account that Gly Cα is relaxed by two directly bonded protons instead of one, this rate constant is rather similar to the *R*_1_ of the β-stranded Ala, implying that the Gly in extended conformations has comparable hydration-induced fast dynamics to the Ala in the β-nanocrystals.

The *R*_1ρ_ rates reveal a so-far unexplored aspect of hydration on the dynamics of the soft segments. In particular, the 63.5 ± 2.1 s^–1^*R*_1ρ_ of the solid Gly Cα stands out from the measured *R*_1ρ_ rates, especially when it is contrasted to the Ala Cα *R*_1ρ_ of 9.9 ± 0.05 s^–1^ of the rigid core. This result suggests that the penetration of water molecules into β-stranded Gly-rich segments substantially increases the chain dynamics on the μs–ms time scale. The slow time scale dynamics of Gly Cα are comparable in the solid and solubilized states since the observed *R*_1ρ_ in the HSQC-based relaxation experiment was only slightly higher than that in the CP-based relaxation experiment (76 ± 13 s^–1^).

The lubricating effect of water is also apparent in the increase of *R*_1ρ_ rates of Ala Cα sites as their conformation transition from β-sheet to α-helix and further to random coil. The β-stranded poly(A) stretches constitute the rigid, hard core of the nanocrystals with minimal μs–ms (and ns) time scale dynamics. The β-nanocrystals are surrounded by a much more flexible, water-exposed α-helical poly(A) shell (*R*_1ρ_ = 32.2 ± 0.5 s^–1^) that is likely in dynamic exchange with the fully solubilized poly(A) segments (*R*_1ρ_ = 43.7 ± 3.2 s^–1^). The dynamic exchange between the solvent-exposed but still immobilized repeat units and the fully solubilized form is further supported by the overall high *R*_1ρ_ rate observed in the CP-based experiment among the Cα and Cβ sites of the Gly-rich soft segment with values between 29.5 and 47.4 s^–1^. In the HSQC-based experiment, an even higher rate is detected for Gln *C*α (*R*_1ρ_ = 85 ± 10 s^–1^), while many other peaks broadened beyond detection. High *R*_1ρ_ and peak broadening are both hallmarks of μs–ms exchange processes.

## Discussion

Spidroins have attracted significant attention in the fields of biotechnology and material science due to their remarkable properties, including the ability to form strong, stable, and tough fibers. However, their expression in alternative hosts like *E. coli* presents challenges, primarily due to the inherent instability and repetitive nature of the encoding DNA sequences, as well as their limited solubility in the expression host.^93^ Consequently, extensive efforts have been dedicated to improving protein production and purification processes, optimizing cloning strategies, and designing genetic circuits to regulate gene expression resources. Despite these endeavors, achieving a high yield in fibrous protein production remains an unresolved issue. Encouragingly, we managed to successfully express a 68 kDa model spidroin protein, N16C, comprising 16 repeat units, with a comparatively high yield in TB medium, without the need for additional metabolic or genetic engineering interventions. The Golden Gate assembly technique employed in constructing the silk gene sequence in this study can be readily applied to generate DNA sequences encoding 32, 64, or even 128 repeat units, thereby enabling the expression of N32C, N64C, or N128C spidroin mimics, respectively. However, it is important to note that producing such large repeat proteins will undoubtedly present several challenges associated with heterologous protein expression.

An extra benefit of being able to express silk-like proteins in *E. coli* is that they can be easily produced in isotopically enriched forms for downstream NMR investigations. Solid-state NMR provides unprecedented insights into the atomic-resolution structure, dynamics, organization, and interaction of semiamorphous semicrystalline systems, like silk. The sensitivity enhancement achieved by uniform ^13^C and ^15^N labeling combined with proton detection and fast solid-state MAS NMR gave us access to a wide range of multidimensional NMR experiments that we customized to focus on specific aspects of the material property. For example, we used two fundamentally different magnetization transfers (cross-polarization and INEPT) to access either the rigid core or the solubilized flexible segments of the hydrated N16C film, and we built these transfer steps into both assignment experiments and spin relaxation measurements. The resulting chemical shifts and relaxation rate constants allowed for the extraction of site-specific information on hydrogen bonding, secondary structure, and nanosecond-to-microsecond time scale dynamics of both rigid and soluble states. Since Ala and Gly are the major constituents of the protein sequence, we focused more on their analysis as they selectively report on the properties of the hard and soft segments.

Since in our designed spidroin sequence, only one type of Ala exists in the repetitive segment, the observed three different sets of Ala resonances must stem from the same poly(A) sequence in different chemical environments. Based on their chemical shifts and relaxation properties, we assigned them to belong to Ala (i) inside the β-nanocrystals, (ii) at the water–protein interface, (iii) and in solution. In the solid-state MAS spectra of native dragline silk fibers, Holland et al. observed β-stranded as well as α-helical Ala and Gly environments, but they associated the separate shifts with sequentially different residues, e.g., with Ala that is part of the poly(A) segments or Ala in GGA elements flanking the poly(A) regions.^19,20,63^

**Figure 8:**
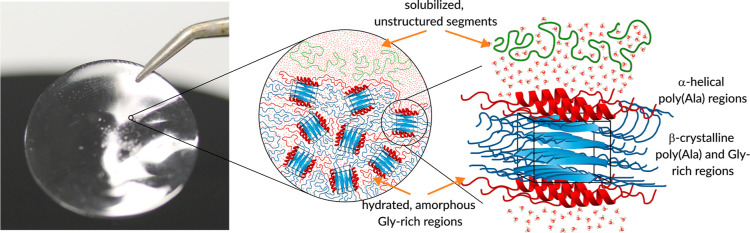
Schematic representation of the conformational states in the hydrated N16C film. The enlargement shows a structural model of the repetitive region in which the poly(Ala) and some of the Gly-rich segments form β-sheets and arrange into randomly oriented β-nanocrystals (blue), whereas the rest of the Gly-rich segments are unordered or adopt a 3_1_-helical structure. At the interface of the β-nanocrystals and amorphous segments, the poly(Ala) repeats form α-helices (red). Some protein chains are fully solubilized and adopt a random coil conformation (green).

The structural and dynamic information on the Ala resonances suggested a model for the repetitive region of the N16C film, where a densely packed β-nanocrystalline core is surrounded by a solvent-exposed layer with α-helical conformation that is in dynamic exchange with fully solubilized repetitive units of N16C, which are highly flexible and unstructured (Figure 8).

The majority of Gly in the soft segment adopted an extended β-sheet conformation and became part of the β-nanocrystals, while a smaller fraction was found in a different conformation that we tentatively associated with the semiextended 3_1_-helical conformation. The large fraction of Gly in β-sheet could be a consequence of casting the film from formic acid. Formic acid is known to initiate β-sheet formation in regenerated *Bombix mori* silk fibroin and in recombinant spider silk films.^69,94−96^ The freshly prepared dry N16C film was brittle but became elastic after isopropanol treatment and incubation in water overnight. Stiffness and brittleness are associated with a high number of β-sheets and long-range order.^84,94^ As water interacts with the ordered β-crystalline structures, the crystallinity decreases and the film becomes more elastic, as demonstrated on *B. mori* fibroin films.^94,97^ In the hydrated N16C film, even though most Gly was incorporated into the rigid β-crystalline domain, it actively contributed to the elasticity of the film as it showed extensive μs time scale flexibility. Our findings align with the results of other X-ray and NMR studies of spider silk fiber that suggested a three-phase model, where the rigid β-crystalline core and the elastic amorphous matrix are interconnected by a semiordered phase.^3,98^

## Conclusions

In this study, we have demonstrated the successful cloning and expression of a designed, synthetic spider dragline silk based on the amino acid sequence of the dragline silk proteins of *Nephila clavipes*. The construct contained all the characteristic building blocks of natural spider silks, including the nonrepetitive N- and CTDs, as well as the repetitive core domains repeated 16 times (N16C), resulting in a final protein size of 68.1 kDa. We achieved the straightforward directional assembly of the modules using Golden Gate assembly, which can be easily utilized to further increase the repetition size.

Additionally, we developed an efficient recombinant protein production and purification strategy and characterized the structure and dynamics of the recombinantly produced, ^13^C,^15^N-labeled spidroin mimic with solution- and solid-state NMR spectroscopy. By analyzing the ^1^H, ^13^C, and ^15^N chemical shifts, we evaluated the secondary structure of the core repetitive domain of N16C dissolved in DMSO-*d*_6_. Furthermore, we analyzed the structure and dynamics of hydrated N16C film cast from formic acid solution using proton-detected solid-state MAS methods, employing both cross-polarization and *J*-coupling-based magnetization transfers. Comparing the solution and solid-state chemical shifts, as well as the ^15^N and ^13^C relaxation rate constants measured in the solid state, we identified three structurally and dynamically distinct segments in the film superstructure. These segments include a rigid, strongly hydrogen-bonded β-nanocrystalline core surrounded by a solvent-exposed, dynamic α-helical shell in exchange with fully solubilized, flexible repeat units with random coil characteristics. These findings highlight the complexity of the hierarchical organization responsible for the remarkable mechanical properties of dragline-silk-mimicking proteins. By harnessing the power of recombinant silk production and advanced spectroscopic techniques, we are one step closer to understanding the correlation between molecular structure and mechanical response of silk-based high-performance materials.

## Supporting Information Available

Supplementary methods on mass spectrometry analysis; sequence alignment; list of primers; protein film analysis; solid-state NMR pulse sequences for *R*_1_ and *R*_1ρ_ measurements; solid-state spectra with assignments; and *R*_1_ and *R*_1ρ_ relaxation decays and derived parameters (PDF)
