# Supplementary material for: Unveiling the Dynamic Self-Assembly of a Recombinant Dragline-Silk-Mimicking Protein
Source: Biomacromolecules. 2024 Feb 12;25(3):1759–74. doi: 10.1021/acs.biomac.3c01239 (PMC10934265; doi:10.1021/acs.biomac.3c01239)
Supplement: Supplementary file 1 — bm3c01239_si_001.pdf [file bm3c01239_si_001.pdf]

Supporting Information: Unveiling the dynamic self-assembly of a recombinant dragline-silk mimicking protein

Dongqing Wu,[†] Anamaria Koscic,[†] Sonja Schneider,[†] Romeo C. A. Dubini,[†,‡] Diana C. Rodriguez Camargo,[†] Sabine Schneider,[†] and Petra Rovó[*,¶,†]

†*Department of Chemistry, Faculty of Chemistry and Pharmacy, Ludwig-Maximilians-Universität München, 81377 Munich, Germany*

‡*Center for Nanoscience (CeNS), Faculty of Physics, Ludwig-Maximilians-Universität München, 80799 Munich, Germany*

¶*Institute of Science and Technology Austria, 3400 Klosterneuburg, Austria*

E-mail: petra.rovo@ist.ac.at

1 Supplementary Methods

1.1 Preparation of N16C sample for mass spectrometry

About 1 mg of the N16C protein was dissolved in 200 μL of 8 M Urea, 10 mM TCEP (tris(2-carboxyethyl)phosphine) and 40 mM CAA (2-chloroacetamide) in 100 mM Tris/HCl pH 8.5 and incubated at 95 °C for 5 min. The reduced and denatured protein was transferred onto 30 kDa cut-off column (Microcon – 30, Centrifugal Filters, Merck) and centrifuged at 14,000 g for 15 min. Subsequently, the filter column was washed twice with 100 μL of 50 mM NH_4HCO_3 in water (ABC buffer) with each centrifugation at 14,000 g for 10 min. The flow-through

was discarded. Next, 100 μL of ABC buffer containing 10 μg trypsin protease (Promega) was added onto the filter columns and mixed for 1 min at 600 rpm and further incubated overnight at 37 °C. The peptides were eluted into a new 1.5 mL tube by centrifugation and two washes each with 40 μL of ABC buffer and centrifugation at 14,000 g for 10 min. The samples were acidified with 1 μL of formic acid (FA) and desalted on SepPak 50 mg C18 columns (Waters). The equilibration and three wash steps were done using 0.5% FA in ddH_2O. Finally, peptides were eluted by 80% acetonitrile (ACN) and 0.5% FA in ddH_2O. Samples were dried on SpeedVac and reconstituted in 100 μL of 1% FA ddH_2O.

1.2 Spectrometer settings

The Orbitrap Eclipse Tribrid Mass Spectrometer was operated in dd-MS2 mode with the following settings: Polarity: positive; MS1 resolution: 240k; MS1 AGC target: standard; MS1 maximum injection time: 50 ms, MS1 scan range: m/z 375-1500; MS2 ion trap scan rate: rapid; MS2 AGC target: standard; MS2 maximum injection time: 35 ms; MS2 cycle time: 1.7 s; MS2 isolation window: m/z 1.2; HCD stepped normalized collision energy: 30%; intensity threshold: $1.0e^4$ counts; included charge states: 2-6; dynamic exclusion: 60 s. Spectra processing was achieved by splitting MS raw files by FAIMS-MzXML-generator and subsequently analyzed with MaxQuant software 1.6.12.0.

2 Supplementary Results

2.1 Sequence alignment

The sequence of the here-engineered and recombinantly produced spidroin protein (N16C) was compared to available spidroin structures in the protein data bank (PDB). Homologous structures for the NTD as well as the CTD, but not for the repetitive core were found. For the NTD, structures of homologous proteins with sequence identities of 30–60%, determined by solution-state NMR as well as X-ray crystallography, are available (Fig. S1), all forming homodimers. Three structures solved by X-ray crystallography to 1.7–2.0 Å resolution were chosen for a detailed comparison, since here information on the water structure within and around the protein is provided. For the CTD only structures determined by NMR are available, with a sequence identity of 30–95%. All structures can be superimposed with an r. m. s. d. of $\sim$1 Å (Fig. S1B and S1F). Within the conserved sequence of the NTD and CTD (Fig. S1), common and partly conserved hydrophobic amino acid residue clusters (Ile, Leu, Val, Ala) were identified and mapped onto the structure of the closest homologous structure available of the NTD spidroin from *Trichonephila clavipes* (PDB code 5IZ2) and CTD from *N. antipodiana* (PDB code 2M0M), (Fig. S1B and G). These hydrophobic clusters are located within the core of the α-helical bundles as well as at the interface of the dimeric structures (Fig. S1). The water structures of the NTD-homologous show that the core and dimer interface is devoid of water molecules (Fig. S1C) and hydrophobic interactions, with some additional hydrogen bonging and salt-bridges are responsible for the molecular architecture. In particular, the CTD-homologous shows very tight hydrophobic packing (Fig. S1G), albeit it needs to be noted that no positional information of water molecules can be derived by NMR. Nevertheless, the hydrophobic nature of the amino acid residues within the structural core makes the presence of water molecules unlikely.

3 Supplementary Tables and Figures

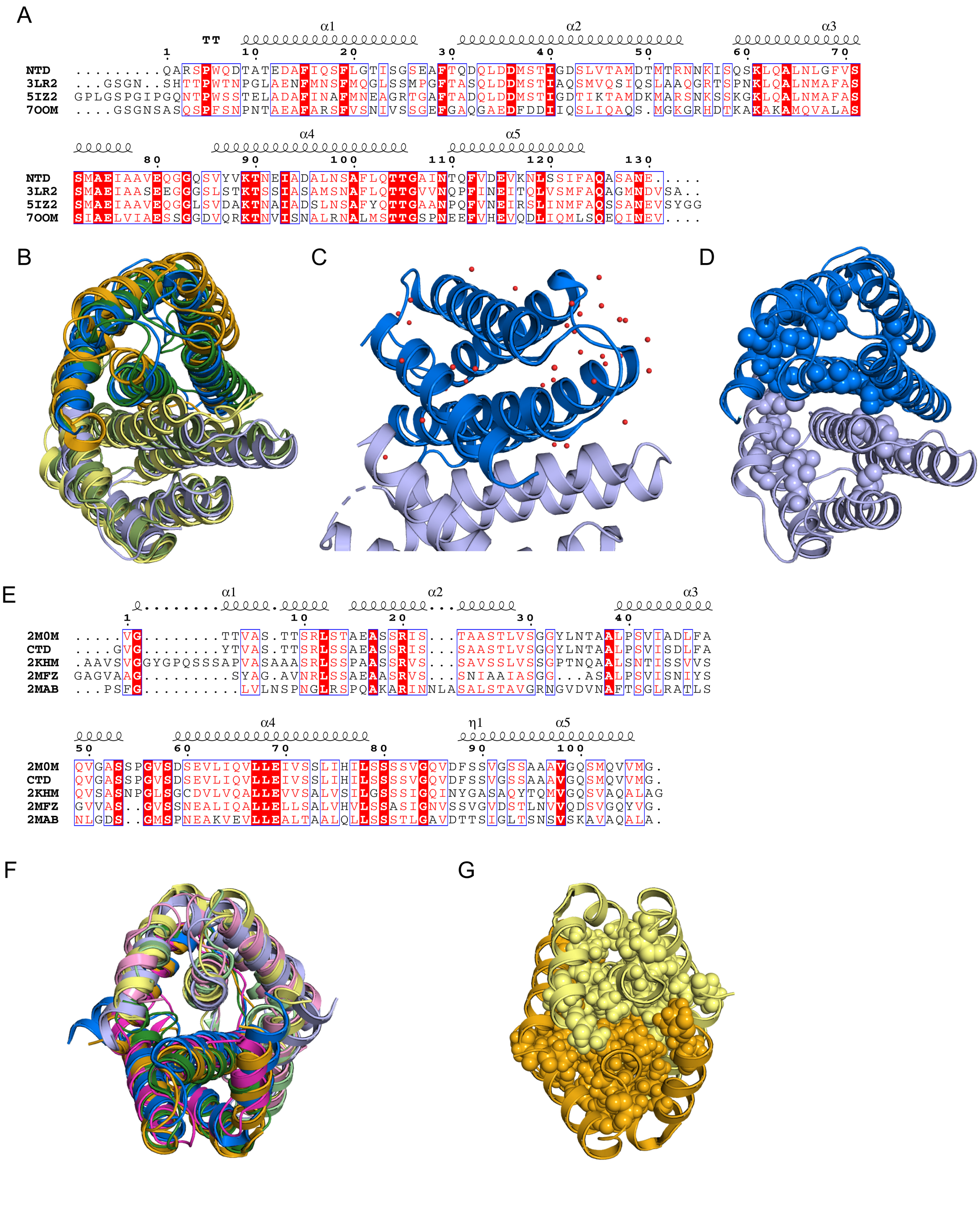

Figure S1: (Caption next page.)

Figure S1: (Previous page.) Comparison of *N. clavipes* MaSp4 NTD and MiSp1 CTD with available spidoin structures identified using HHPRED.[S1] A) Sequence alignment of the sequence of the NTD with homologous from *Euprosthenops australis* (PDB code 3LR2, 43% sequence identity) and *Trichonephila clavipes* MaSpi1A (PDB code 5IZ2, 57% sequence identity) and *T. clavipes*; Flagelliform Spidroin variant 1 (PDB code 7OOM, 32% sequence identity). These structures have been determined using X-ray crystallography to 1.7-2Å resolution, thus providing information about the water structure within the protein. B) Structural superposition of the homodimeric structures (PDB code 3LR2 green/light green, PDB code 5IZ2 blue/ light blue, PDB code 7OOM golden/yellow) highlighting the similar molecular architecture. C) Water structure (red spheres) within the *T. clavipes* MaSpi1A (PDB code 5IZ2) homodimer, highlighting the water-devoid, hydrophobic core of the architecture. D) Hydrophobic Ile-Leu-Val clusters within *T. clavipes* MaSpi1A shown as spheres. E) Sequence alignment of the sequence of the CTD with homologous from *N. antipodiana* Minor ampullate fibroin 1 (PDB code 2M0M, 95% sequence identity), *Araneus diadematus* Fibroin-3 (PDB code 2KHM, 47% sequence identity), *Araneus ventricosus* Minor ampullate spidroin (PDB code 2MFZ, 46% sequence identity), *N. antipodiana* aciniform spidroin (PDB code 2MAB, 31% sequence identity). F) Superposition highlighting the similar molecular architecture of the homodimeric structures. (PDB code 2KHM blue/light blue; PDB code 2MOM golden/yellow, PDB code 2MFZ green/light green, PDB code 2MAB pink/light pink). G) Hydrophobic Ile-Leu-Val clusters within *N. antipodiana* Minor ampullate fibroin 1 (PDB code 2M0M) are shown as spheres, highlighting the tight packing within the core of the homodimer. Sequence alignments were done with ClustalX[S2] and annotated using the Espript server[S3]

Table S1: Overview of the primers used during the Golden Gate Assembly. Bold and underlined nucleotides represent the recognition site for the type IIs endonucleases (*BseR*I and *Bsa*I) and the corresponding restriction sites, respectively.

**Primer**	**Orientation**	**Sequence (5'–3')**
Step 1 insert 1	Forward	GAAGA**GAGGAG**ACATATGGGTCAGCAGG
	Reverse	CCCTC**GAGGAG**TAGTTATTCCACGCCGC
Step 1 Insert 2	Forward	TAAGAA**GAGGAG**ATACATATGGGTCAGCA
	Reverse	CTAGTG**GAGGAG**CCGTGATGCAGGAA
Step 1 Backbone	Forward	AGCACC**GAGGAG**ACCACCACTGAGATCCG
	Reverse	CCGCGC**GAGGAG**AGGCCGCTACCGTGAT
Step 2a Insert	Forward	AATTAA**GGTCTC**ACCACCACCAAGCGCGTAGCCCG
	Reverse	ACGTAT**GGTCTC**AATCAAACTTCGTTCGCGCTC
Step 2a Backbone	Forward	TACGTAA**GGTCTC**ATGATAAGAGATCCGGCTGCTAACAAAG
	Reverse	TATATA**GGTCTC**AGTGGTGGTGGTGGTGCATGGTATATCTCCTTCTTAAA
Step 2b Insert	Forward	ATGCAC**GGTCTC**AATGCATCTAGATGGCGTGGGTTACA
	Reverse	ATGTAA**GGTCTC**ATGGTGGTGGTGGCCCATAACCACTTGC
Step 2b Backbone	Forward	AATTGG**GGTCTC**AACCACCACCACCACTAATGAGTGAGATCCGGCTGCTAA
	Reverse	ATGCAT**GGTCTC**AGCATCATGGTATATCTCCTTCTTAAAGTTAAACAAA
Step 3 Backbone + NTD	Forward	AACAA**GAGGAG**AAAGGAAGCTGAGTTGGC
	Reverse	AGCAGC**GAGGAG**TCTTATCATACTTCGTTC
Step 3 16mer	Forward	AGCCA**GAGGAG**TCATCATCTAGGTCAGC
	Reverse	GTACTA**GAGGAG**GTGTTATTGCACGCCG
Step 3 CTD	Forward	GAAGG**GAGGAG**ACCATGATGCATCTAGATG
	Reverse	GTTGTG**GAGGAG**GTGGTGGTAGCCCAT

Table S2: Sequence and molecular weight found by mass spectrometric analysis

**Primary sequence**	**Position**	**M_w (Da)**
LQALNLGFVSSMAEIAAVEQGGQSVYVK	NTD (69-96)	2908.50
NLSSIFAQASANEVGQQGPGGSGPAAAAAAAGGSGQGGYGGLGR	NTD and Repetitive motive (125-168)	3872.84
GGVGQQGPGGSGPAAAAAAAGGSGQGGYGGLGR	Repetitive motive	2654.25
GGVHLDGVGYTVASTTSR	CTD (664-681)	1775.88

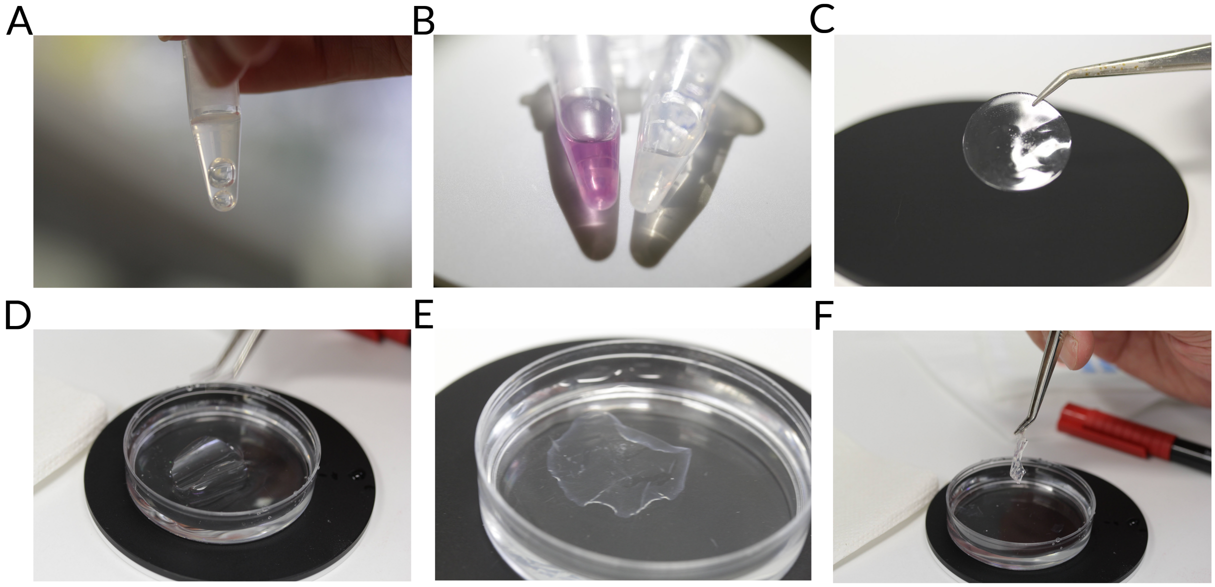

Figure S2: (A) N16C protein in formic acid forms a gelatinous solution. (B) The solution of N16C in formic acid turns pink overnight at room temperature while the solution of S16C in formic acid shows no color change. (C) Freshly cast N16C film after incubation with isopropanol for 2 hours. (D) The N16C film is stable in water, the softness and elasticity increase dramatically after sufficient wetting. (E) The S16 film swells immediately in water and is starting to disintegrate. (F) The S16 film loses its shape when it gets wet.

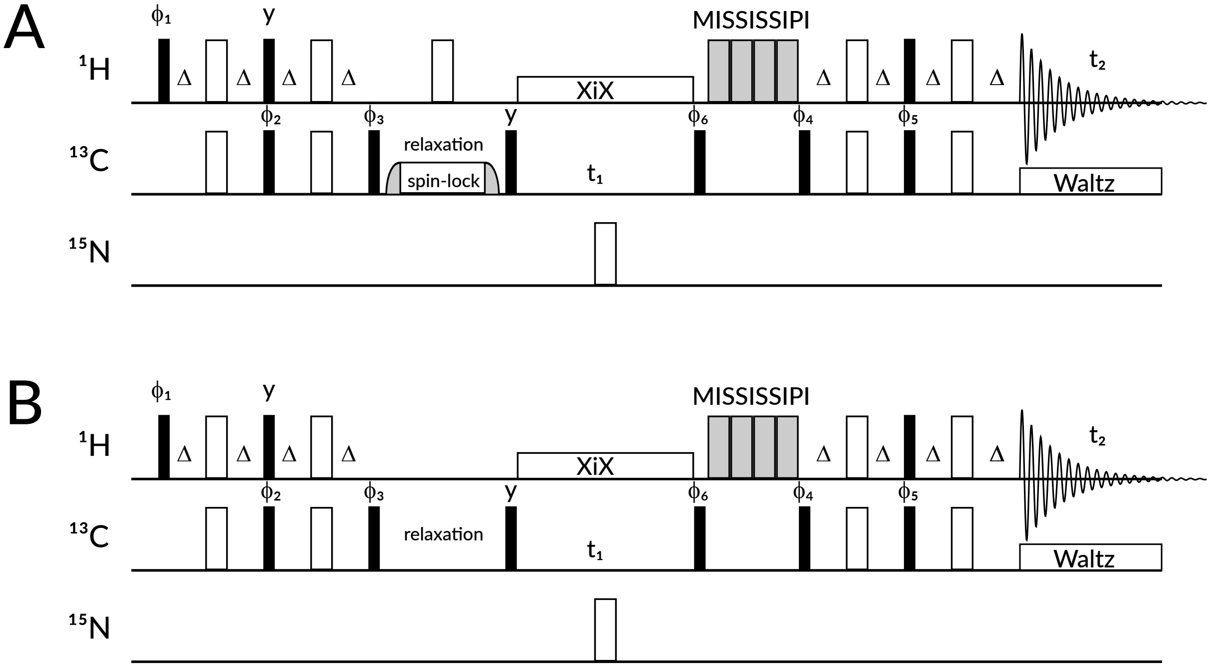

Figure S3: Pulse sequences for the measurement of ^{13}C $R_{1\rho}$ (A) and R_1 (B) using scalar-based INEPT for magnetization transfer. All narrow solid bars are 90° pulses along the x-axis, unless indicated otherwise. Wide open bars are 180° pulses. The train of proton pulses for water suppression follows the MISSISSIPI scheme. Decoupling of ^{1}H during t_1 evolution is achieved via a 12.5 kHz XiX scheme. Decoupling of ^{13}C during t_2 is achieved via a 10 kHz Waltz-16 sequence. The delay Δ is set to less than $1/(4J_{CH})$ to reduce signal loss due to relaxation. Phase cycling is: $\phi_1 = x, -x$, $\phi_2 = 2(x), 2(-x)$, $\phi_3 = 8(y), 8(-y)$, $\phi_4 = 4(x), 4(-x)$, $\phi_5 = 4(y), 4(-y)$. The receiver phase is $\phi_{\text{rec}} = x, -x, -x, x, x, -x, -x, x, -x, x, x, -x, -x, x, x, -x$. Quadrature detection in t_1 is achieved by alternating ϕ_6 following the t_1 evolution according to States-TPPI scheme. A series of relaxation measurements were recorded by incrementing the relaxation delay. $R_{1\rho}$ measurements were performed with an on-resonance spinlock. Even spin-locking was achieved by adiabatic excitation. The non-rectangular gray pulses bracketing the spin-lock pulse are adiabatic half-passage pulses with a shape of tanh/tan, a sweep width of 100 kHz, and a duration of 4 ms.

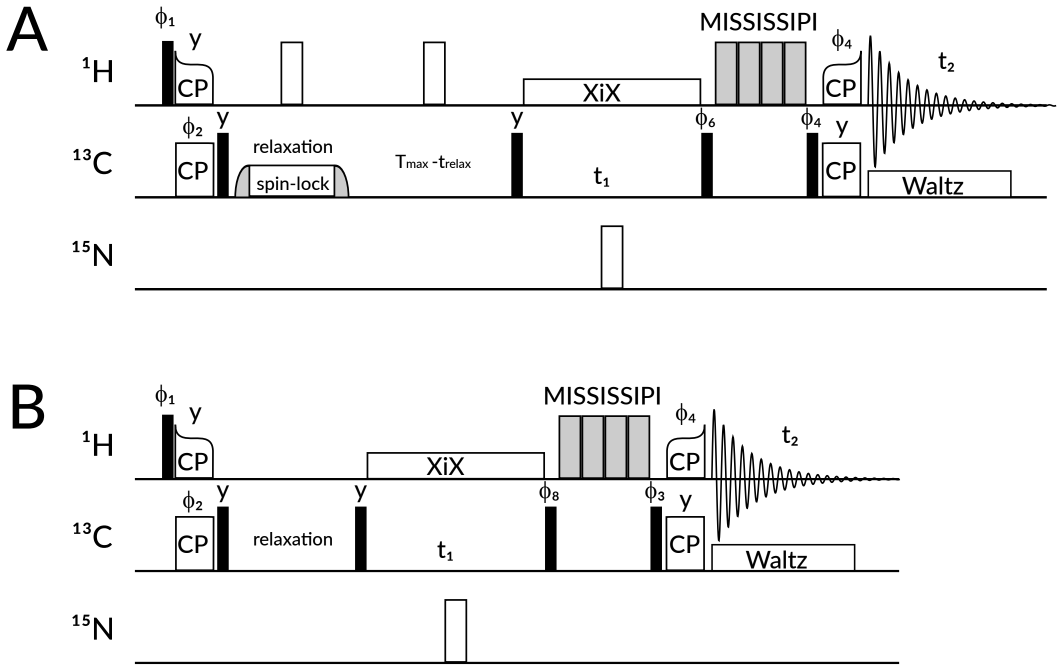

Figure S4: Pulse sequences for the measurement of ^{13}C $R_{1\rho}$ (A) and R_1 (B) using dipolar-based cross-polarization for magnetization transfer. All narrow solid bars are 90° pulses along the x-axis, unless indicated otherwise. Wide open bars are 180° pulses. The train of proton pulses for water suppression follows the MISSISSIPI scheme. Decoupling of ^{1}H during t_1 evolution is achieved via a 12.5 kHz XiX scheme. Decoupling of ^{13}C during t_2 is achieved via a 10 kHz Waltz-16 sequence. Cross polarization between ^{1}H and ^{13}C was achieved with a rectangular-shaped pulse on the ^{13}C channel and a 75% tangential-shaped pulse on the ^{1}H channel with a duration of 500 μs matching the zero-quantum Hartman-Hahn condition. Phase cycling is: $\phi_1 = x, -x$, $\phi_2 = 8(x), 8(-x)$, $\phi_3 = 2(x), 2(-x)$, $\phi_4 = 4(x), 4(-x)$. The receiver phase is $\phi_{\mathrm{rec}} = x, -x, -x, x, x, -x, -x, x, -x, x, x, -x, -x, x, x, -x$. Quadrature detection in t_1 is achieved by alternating ϕ_8 following the t_1 evolution according to States-TPPI scheme. A series of relaxation measurements were recorded by incrementing the relaxation delay. $R_{1\rho}$ measurements were performed with an on-resonance spinlock. Even spin-locking was achieved by adiabatic excitation. The non-rectangular gray pulses bracketing the spin-lock pulse are adiabatic half-passage pulses with a shape of tanh/tan, a sweep width of 100 kHz, and a duration of 4 ms.

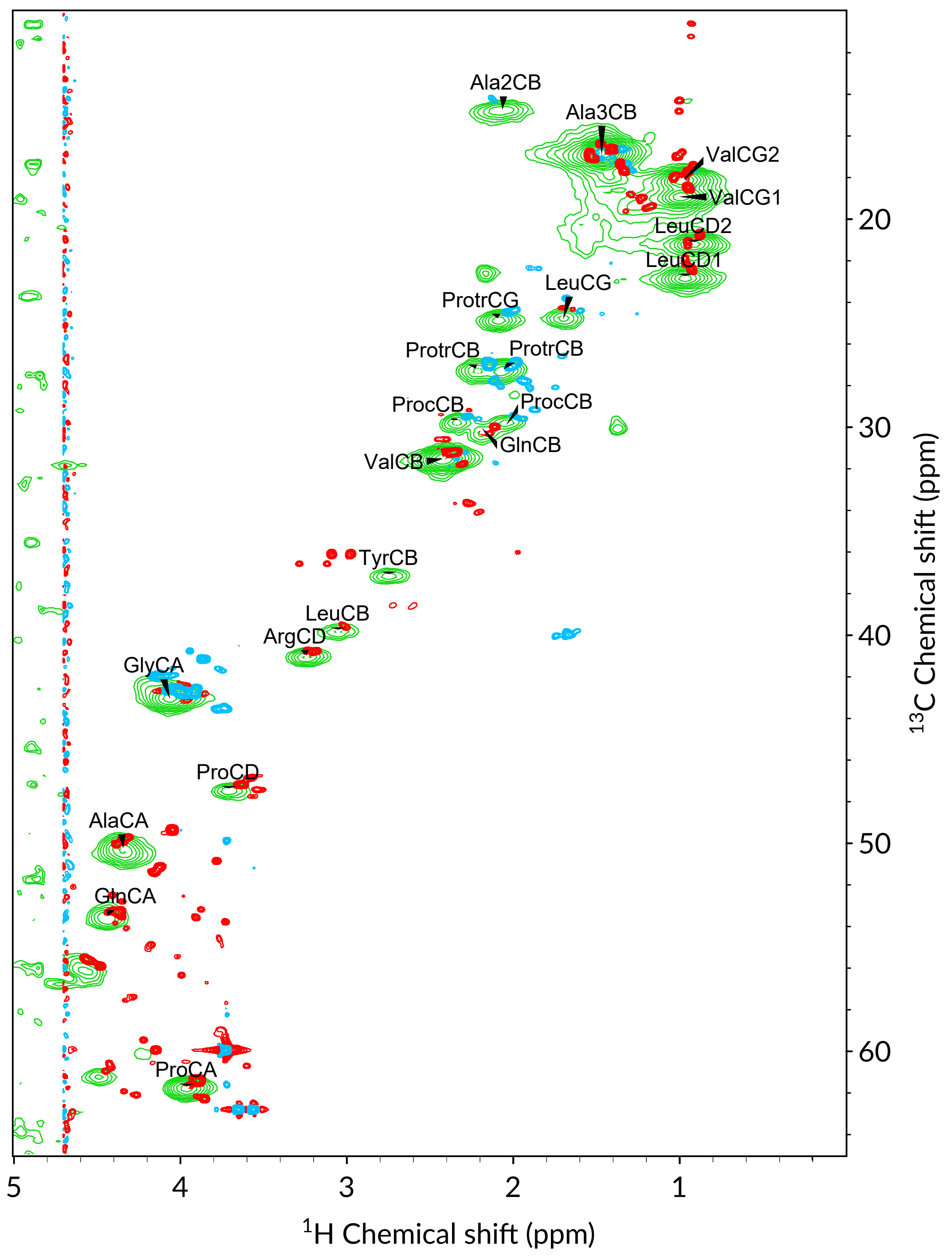

Figure S5: Overlay of the solid-state (green) and solution-state (red and blue) ^{1}H-^{13}C HSQC spectra of N16C. Assignments are shown for the solid-state spectrum.

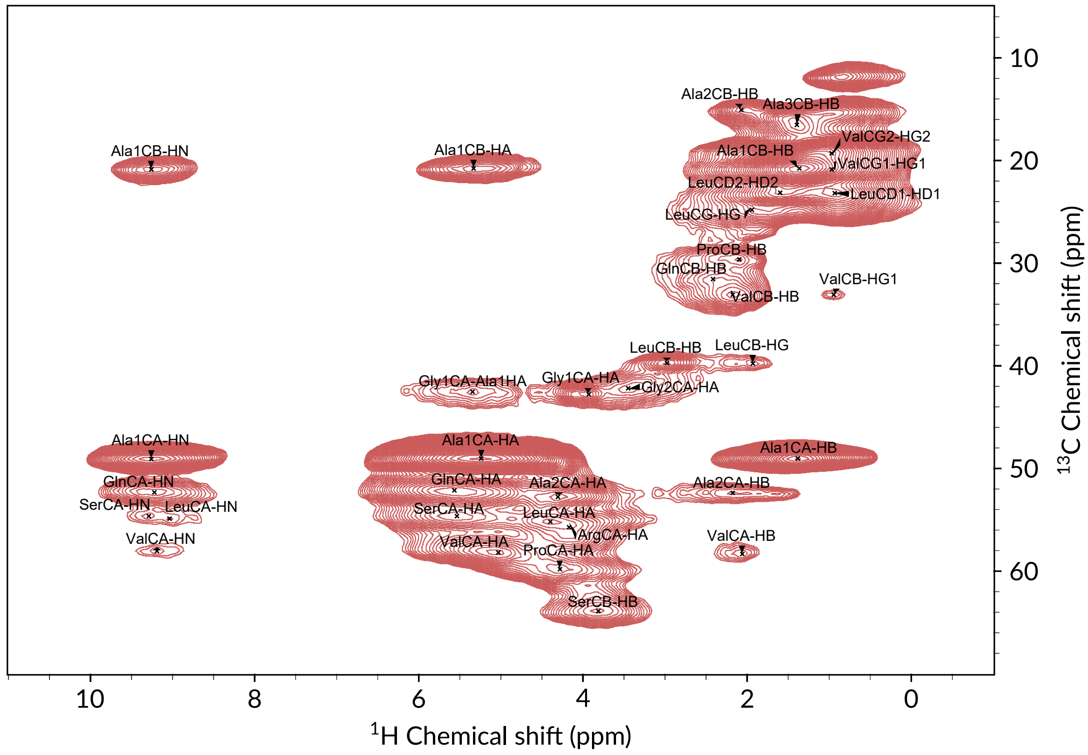

Figure S6: ^{1}H-^{13}C CP-based HCH spectrum of N16C showing the amino-acid-specific assignment. Ala1, Ala2, and Ala3 refer to the alanines in β-sheet, α-helix, or in random coil conformations. Gly1 and Gly2 refer to the glycines in β-sheet, or in random coil conformations, respectively.

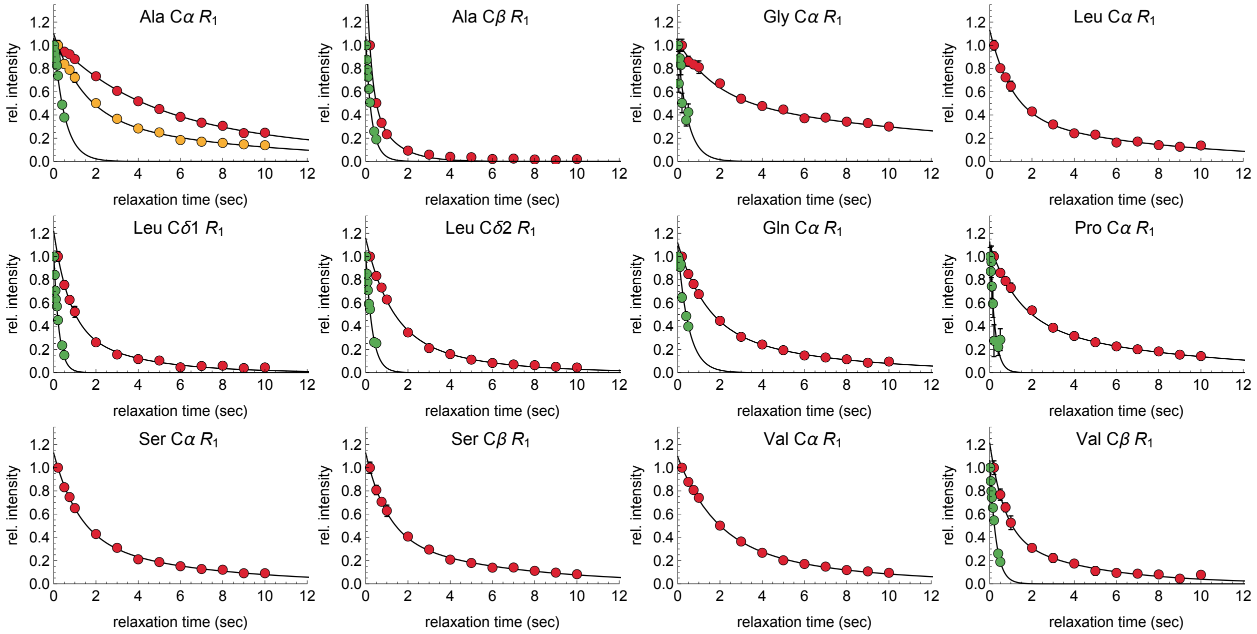

Figure S7: ^{13}C R_1 relaxation decays of all sites that were observable in the solid-state CP or HSQC-based relaxation experiments. Red and green data points were derived from the CP- and HSQC-based experiments, respectively. For Ala Cα and Cβ the red, yellow, and green symbols represent the sites that belong to the extended, helical, and random coil conformations. Solid lines are the best biexponential (CP-based) or monoexponential (HSQC-based) fits of the decays.

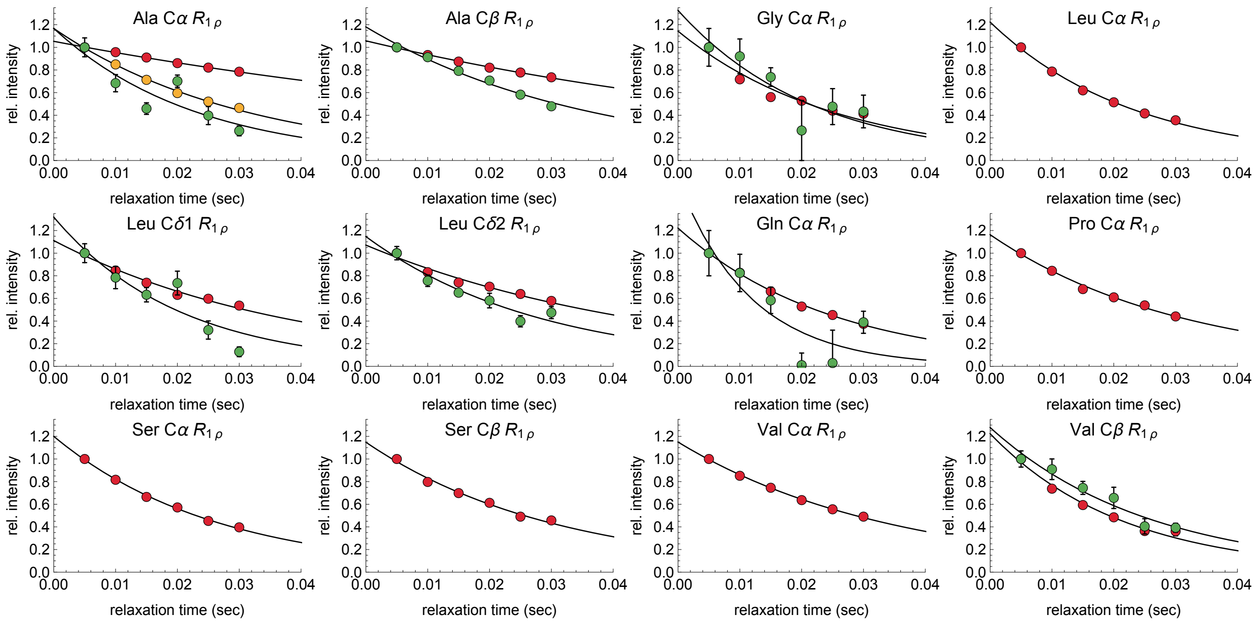

Figure S8: ^{13}C $R_{1\rho}$ relaxation decays of all sites that were observable in the solid-state CP or HSQC-based relaxation experiments. Red and green data points were derived from the CP- and HSQC-based experiments, respectively. For Ala Cα and Cβ the red, yellow, and green symbols represent the sites that belong to the extended, helical, and random coil conformations. Solid lines are the best monoexponential fits of the decays.

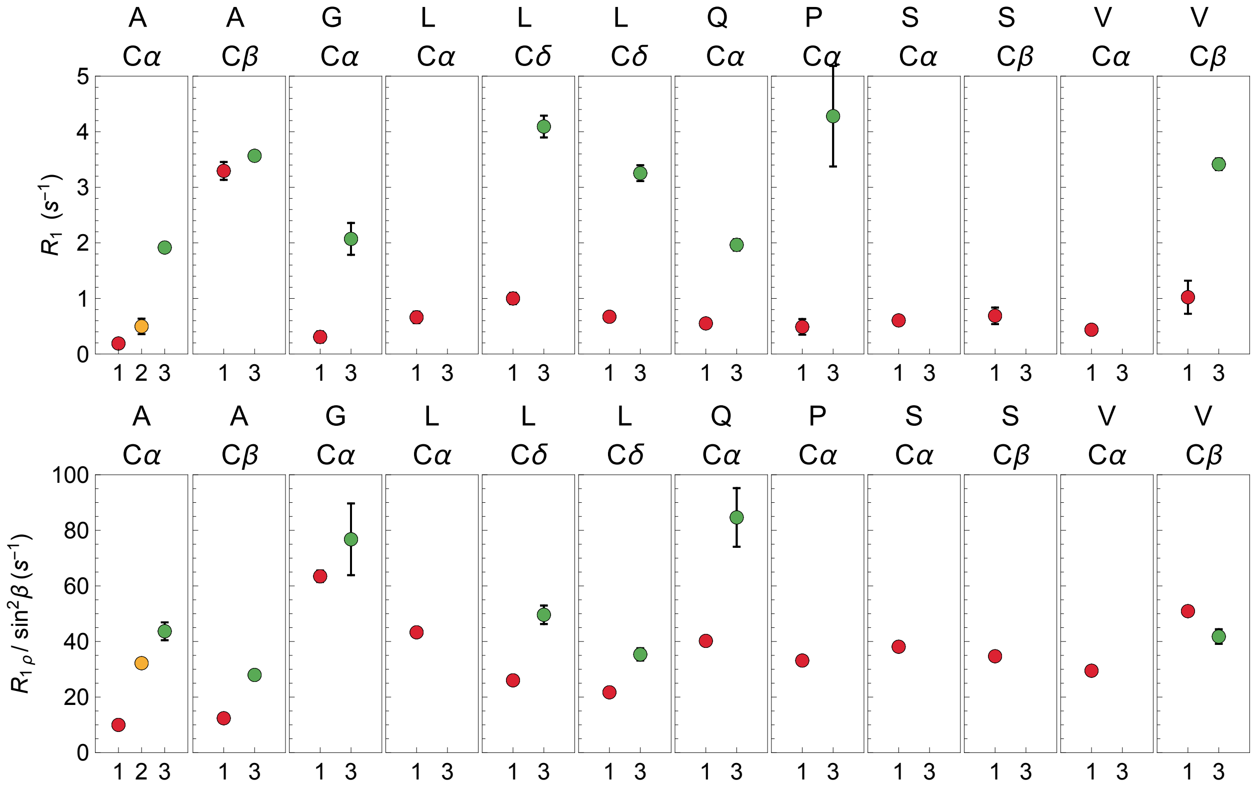

Figure S9: R_1 (upper row) and $R_{1\rho}/\sin^2\beta$ (bottom row) relaxation rate constants of Cα, Cβ, or Cδ sites of N16C. Red (1), yellow (2), and green (3) symbols represent the sites that were observed in the CP-based (1 and 2) and in the HSQC-based (3) experiments. For Ala Cα the rates were determined for the extended (red, 1), α-helical (yellow, 2), and random coil (green, 3) conformations.

Table S3: Fitted parameters obtained from the HSQC-based ^{13}C R_1 experiment. R_1^{mono} and R_1^{bi} correspond to the rate constants of the mono-exponential and biexponential fits, respectively. Ala1, Ala2, and Ala3 refer to the alanines in β-sheet, α-helix, or in random coil conformations. Gly3 refers to the glycines in solubilized, random coil conformation.

**Atom name**	R_1^{mono} / s^{-1}	R_1^{bi} / s^{-1}	χ^2 **mono**	χ^2 **bi**	**F-test**	**selected model**
Ala3 Cα	1.92 ± 0.046	1.92 ± 0.046	0.198	0.198	0.475	mono
Ala3 Cβ	3.56 ± 0.026	3.91 ± 0.46	0.212	0.103	0.253	mono
Ala2 Cβ	0.81 ± 0.077	0.84 ± 0.28	0.216	0.216	0.476	mono
Gln Cα	1.96 ± 0.103	1.96 ± 0.10	0.693	0.693	0.475	mono
Gln Cβ	1.75 ± 0.207	3.67 ± 2.24	0.812	0.913	0.426	mono
Gly3 Cα	2.07 ± 0.289	2.34 ± 1.12	1.41	1.41	0.478	mono
Leu Cδ1	4.09 ± 0.196	9.56 ± 9.16	0.357	11.5	0.0003	mono
Leu Cδ2	3.25 ± 0.143	4.66 ± 3.30	0.341	2.22	0.022	mono
Pro Cα	4.28 ± 0.904	5.16 ± 2.91	0.751	0.739	0.482	mono
Val Cβ	3.41 ± 0.105	3.44 ± 0.36	0.044	0.043	0.486	mono
Val Cγ	3.58 ± 0.046	4.37 ± 1.91	0.268	2.90	0.007	mono

Table S4: Fitted parameters obtained from the CP-based ^{13}C R_1 experiment. R_1^{mono} and R_1^{bi} correspond to the rate constants of the mono-exponential and biexponential fits, respectively. Ala1, Ala2, and Ala3 refer to the alanines in β-sheet, α-helix, or in random coil conformations. Gly1 refers to the glycines in β-sheet conformation.

**Atom name**	R_1^{mono} / s^{-1}	R_1^{bi} / s^{-1}	χ^2 **monoexp**	χ^2 **biexp**	**F-test**	**selected model**
Ala1 Cα	0.160 ± 0.001	0.190 ± 0.003	1.96	0.591	0.0413	bi
Ala1 Cβ	1.94 ± 0.049	3.29 ± 0.161	2.55	0.955	0.0757	bi
Ala2 Cα	0.280 ± 0.011	0.497 ± 0.139	1.88	0.142	0.0003	bi
Ala3 Cβ	1.45 ± 0.110	2.51 ± 0.484	1.31	0.349	0.0282	bi
Gln Cα	0.351 ± 0.008	0.550 ± 0.051	2.65	0.084	0.00001	bi
Gly1 Cα	0.138 ± 0.007	0.307 ± 0.100	1.36	0.176	0.0023	bi
Leu Cα	0.313 ± 0.013	0.660 ± 0.103	2.88	0.168	0.0001	bi
Leu Cδ1	0.677 ± 0.049	0.100 ± 0.101	1.27	0.204	0.0052	bi
Leu Cδ2	0.512 ± 0.021	0.670 ± 0.049	1.29	0.156	0.0018	bi
Pro Cα	0.256 ± 0.008	0.488 ± 0.141	1.78	0.092	0.00006	bi
Ser Cα	0.361 ± 0.014	0.605 ± 0.078	2.33	0.092	0.00002	bi
Ser Cβ	0.367 ± 0.023	0.688 ± 0.149	1.81	0.071	0.00002	bi
Val Cα	0.316 ± 0.010	0.436 ± 0.047	1.19	0.050	0.00003	bi
Val Cβ	0.524 ± 0.054	1.021 ± 0.298	1.51	0.154	0.00098	bi

Table S5: Fitted parameters obtained from the HSQC-based ^{13}C $R_{1\rho}$ experiment. O2P refers to the carrier position during the spin-lock, $\omega_e/2\pi$ and β_e are the effective irradiation field and effective angle. Ala1, Ala2, and Ala3 refer to the alanines in β-sheet, α-helix, or in random coil conformations. Gly3 refers to the glycines in solubilized, random coil conformation.

**Atom name**	O2P (ppm)	$R_{1\rho}$ / $\mathbf{s}^{-1}$	$\omega_e/2\pi$ / kHz	β_e / deg.	$R_{1\rho}/\sin^2\beta_\mathbf{e}$ / $\mathbf{s}^{-1}$
Ala3 Cα	53	43.52 ± 6.41	7.51	86.7	43.68 ± 3.21
Ala3 Cβ	20	27.81 ± 0.54	7.52	85.9	27.95 ± 0.27
Ala2 Cβ	20	41.43 ± 5.85	7.55	83.3	42.00 ± 2.97
Gln Cα	53	84.62 ± 21.1	7.50	-88.9	84.7 ± 10.5
Gln Cβ	20	62.00 ± 13.0	7.72	-76.2	65.7 ± 6.8
Gly3 Cα	20	46.0 ± 16.0	6.46	-50.76	77.0 ± 13.0
Leu Cδ1	20	49.37 ± 6.62	7.52	-86.0	49.60 ± 3.33
Leu Cδ2	20	35.28 ± 4.42	5.00	-87.4	35.35 ± 2.21
Val Cβ	20	38.86 ± 4.88	7.78	-74.7	41.78 ± 2.62
Val Cγ	20	26.81 ± 1.49	7.50	87.6	26.86 ± 0.75

Table S6: Fitted parameters obtained from the CP-based ^{13}C $R_{1\rho}$ experiment. O2P refers to the carrier position during the spin-lock, $\omega_e/2\pi$ and β_e are the effective irradiation field and effective angle. Ala1, Ala2, and Ala3 refer to the alanines in β-sheet, α-helix, or in random coil conformations. Gly1 refers to the glycines in β-sheet conformation.

**Atom name**	O2P (ppm)	$R_{1\rho}$ / $\mathbf{s}^{-1}$	$\omega_e/2\pi$ / kHz	β_e / deg.	$R_{1\rho}/\sin^2\beta_\mathbf{e}$ / $\mathbf{s}^{-1}$
Ala1 Cα	53	9.90 ± 0.05	7.53	84.8	9.98 ± 0.02
Ala1 Cβ	20	12.38 ± 0.13	7.50	-88.9	12.37 ± 0.06
Ala2 Cα	53	32.19 ± 0.96	7.50	89.8	32.19 ± 0.48
Ala3 Cβ	20	29.43 ± 1.18	7.52	85.1	29.64 ± 0.59
Gln Cα	53	40.20 ± 0.61	7.50	89.4	40.20 ± 0.30
Gly1 Cα	20	39.25 ± 2.54	6.36	-51.8	63.47 ± 2.06
Leu Cα	53	43.19 ± 1.20	7.51	-87.1	43.30 ± 0.60
Leu Cδ1	20	25.87 ± 0.79	7.52	-85.7	26.02 ± 0.40
Leu Cδ2	20	21.43 ± 0.44	5.03	-83.5	21.71 ± 0.22
Pro Cα	53	32.32 ± 1.01	7.59	-80.9	33.14 ± 0.52
Ser Cα	53	38.06 ± 0.84	7.50	-87.7	38.11 ± 0.42
Ser Cβ	53	32.50 ± 1.62	7.75	-75.4	34.69 ± 0.87
Val Cα	53	29.02 ± 0.73	7.56	-82.8	29.48 ± 0.37
Val Cβ	20	46.60 ± 2.38	7.84	-73.0	50.92 ± 1.30

References

(S1) Zimmermann, L.; Stephens, A.; Nam, S.-Z.; Rau, D.; Kübler, J.; Lozajic, M.; Gabler, F.; Söding, J.; Lupas, A. N.; Alva, V. A Completely Reimplemented MPI Bioinformatics Toolkit with a New HHpred Server at its Core. *Journal of Molecular Biology* **2018**, *430*, 2237–2243.

(S2) Larkin, M.; Blackshields, G.; Brown, N.; Chenna, R.; McGettigan, P.; McWilliam, H.; Valentin, F.; Wallace, I.; Wilm, A.; Lopez, R.; Thompson, J.; Gibson, T.; Higgins, D. Clustal W and Clustal X version 2.0. *Bioinformatics* **2007**, *23*, 2947–2948.

(S3) Robert, X.; Gouet, P. Deciphering key features in protein structures with the new ENDscript server. *Nucleic Acids Research* **2014**, *42*, W320–W324.
